# The response and recovery of the *Arabidopsis thaliana* transcriptome to phosphate starvation

**DOI:** 10.1186/1471-2229-12-62

**Published:** 2012-05-03

**Authors:** Jongchan Woo, Cameron Ross MacPherson, Jun Liu, Huan Wang, Takatoshi Kiba, Matthew A Hannah, Xiu-Jie Wang, Vladimir B Bajic, Nam-Hai Chua

**Affiliations:** 1 Laboratory of Plant and Molecular Biology, The Rockefeller University, New York, 10065, NY, USA; 2 Bayer Crop Science, Technologiepark 38, 9052, Ghent, Belgium; 3 State Key Laboratory of Plant Genomics, Institute of Genetics and Developmental Biology, Chinese Academy of Sciences, Beijing, 100101, China; 4 Computational Bioscience Research Center (CBRC), King Abdullah University of Science and Technology (KAUST), Thuwal, Kingdom of Saudi Arabia

**Keywords:** Phosphate starvation, Response and recovery, Roots and shoots, Organ specific, Whole seedling, Initial, Persistent, and Latent expression patterns, Functional analysis, Comparative analysis with AtGenExpress, Micro-array and tiling-array, Hydroponic culture

## Abstract

**Background:**

Over application of phosphate fertilizers in modern agriculture contaminates waterways and disrupts natural ecosystems. Nevertheless, this is a common practice among farmers, especially in developing countries as abundant fertilizers are believed to boost crop yields. The study of plant phosphate metabolism and its underlying genetic pathways is key to discovering methods of efficient fertilizer usage. The work presented here describes a genome-wide resource on the molecular dynamics underpinning the response and recovery in roots and shoots of *Arabidopsis thaliana* to phosphate-starvation.

**Results:**

Genome-wide profiling by micro- and tiling-arrays (accessible from GEO: GSE34004) revealed minimal overlap between root and shoot transcriptomes suggesting two independent phosphate-starvation regulons. Novel gene expression patterns were detected for over 1000 candidates and were classified as either initial, persistent, or latent responders. Comparative analysis to AtGenExpress identified cohorts of genes co-regulated across multiple stimuli. The hormone ABA displayed a dominant role in regulating many phosphate-responsive candidates. Analysis of co-regulation enabled the determination of specific versus generic members of closely related gene families with respect to phosphate-starvation. Thus, among others, we showed that *PHR1-*regulated members of closely related phosphate-responsive families (*PHT1;1*, *PHT1;7–9*, *SPX1-3*, and *PHO1;H1*) display greater specificity to phosphate-starvation than their more generic counterparts.

**Conclusion:**

Our results uncover much larger, staged responses to phosphate-starvation than previously described. To our knowledge, this work describes the most complete genome-wide data on plant nutrient stress to-date.

## Background

Phosphorus is one of the most important macronutrients in crop fertilizers [1,2]. Although it is required in only small quantities [3] most crop plants require inorganic forms of phosphorous such as phosphate (P_i_) that is not readily available in many soil conditions [4,5]. Because of P_i_-limiting stress, P_i_ fertilizers are used in agriculture to elevate soil P_i_ concentrations to increase crop productivity [4]. However, over-application of P_i_ fertilizers has led to run-off of excess P_i_ into waterways damaging ecosystems [6-8]. Therefore, there is economic and ecological impetus underlying research into plant P_i_ signaling pathways with the expectation that results from such work may help to develop crop plants with enhanced P_i_ use efficiency, thereby mitigating the unwarranted, excessive application of P_i_ fertilizers.

Whereas many researchers have studied P_i_ signaling in the model plant *Arabidopsis thaliana,* it is only recently that high-throughput technologies have been applied to address this issue [9-14]. To date, a limited amount of genome-wide data is available and there is no one dataset that can serve as a reference for comparison in studies on P_i_-signaling. Our ability to relate one genome-wide study to another is, therefore, constrained by insufficient data. Here, we aimed to construct a referable dataset by recording and characterizing the response and recovery of *Arabidopsis thaliana*’s whole genome transcriptome to P_i_ starvation (P_i_^starv^) at a higher resolution than previously reported.

Previous studies have implicated common sets of differentially regulated genes during related P_i_^starv^ treatments but a large degree of variation exists among independent studies (see Additional file 1). Several factors may contribute to this observed variation: (1) Different methodologies were used to manipulate P_i_ concentrations in the local environment; (2) Different tissues, organs, and plant species were studied, that may implement different P_i_-sensing and gene response circuitry; (3) Previous work did not analyze independent responses of roots and shoots on a genome-wide scale; (4) Due to natural P_i_-reservoirs in plant samples it is difficult to determine the duration of starvation treatment; (5) There is a lack of a comprehensive study serving as a basic reference.

To address the above-mentioned issues we aimed to not only identify novel genetic components in the Arabidopsis P_i_^starv^ response but also provide results that can serve as a reference for future investigations. To this end, we designed experiments to evaluate differential gene expression separately in roots and shoots of plants subjected to P_i_^starv^ (“response” phase) followed by return to replete conditions (“recovery” phase). In total, we analyzed 18 micro- and 18 tiling-arrays consisting of the following sample structure: 3 biological replicates sampled from 2 organs (roots and shoots), each exposed to 3 conditions (mock (P_i_^mock^), starvation (P_i_^starv^), and replete (P_i_^replete^)). Gene response was evaluated by contrasting P_i_^starv^ against P_i_^mock^, whereas gene recovery was calculated by contrasting P_i_^replete^ versus P_i_^starv^ samples. In this way, we aimed to address the first three issues by providing a common reference for the analysis of organ-specific response and recovery. To address (4) we provided molecular evidence to demonstrate that genes did indeed respond to, and subsequently recovered from, P_i_^starv^. We addressed (5), in part, as our experimental model generated a high-quality dataset by analyzing both response-and-recovery data from plants conditioned in the same environment using both the ATH1 micro-array and the tiling-array platform for transcriptome analysis.

Our data indicated a dramatic difference in the molecular responses of roots and of shoots both during and post P_i_^starv^. We showed that significantly different regulons are involved in P_i_^starv^ in both a time and organ dependent manner. Furthermore, in comparative studies with micro-array data generated by the AtGenExpress initiative we developed a method for the determination of specific versus generic members of closely related gene families with respect to P_i_^starv^. Being a genome-wide study, we identified many genes previously unknown to respond to and/or recover from P_i_^starv^. To further verify the specificity of their functions we conducted literature surveys on possible co-regulation by other stresses of many candidates. We believe that our work collectively describes the highest resolution of genome-wide data available to the community to date. The accession code, GSE34004, may be used to access the micro- and tiling-array data from the Gene Expression Omnibus (GEO).

## Results

### Experimental design and quality assessment

**Figure 1:**
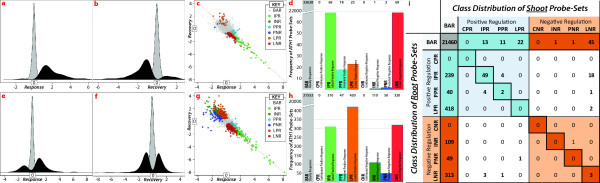
**The distribution of probe-sets across tissues and response-and-recovery classes.** Subfigures (**a**), (**b**), (**e**), and (**f**) display the relative distribution (no y-axis) of fold-change values (log_2_) for significantly (black curve) and non-significantly (grey curve) regulated probe-sets in shoot (**a, b**) and root (**e, f**) for both response (**a, e**) and recovery (**b, f**). (**c, g**) Dot plot of fold-change values for response (x-axis) against recovery (y-axis) from shoot (**c**) and root (**g**) samples. Probe-sets tend to recovery as is evident by their trend (line of full recovery, dotted red line, y = −x). Significantly regulated probe-sets are colored according to their response-and-recovery classification (Table 1). (**d, h**) Histogram of probe-set counts (y-axis) according to response-and-recovery classes (Table 1) for shoot (**d**) and root (**h**). (**i**) Table showing the intersection between shoot (columns) and root (rows) response-and-recovery classes. The top leftmost cell being a count of probe-sets neither responsive in shoot nor root. The first column and row being counts of loci uniquely regulated by root and shoot, respectively. The diagonal being counts of ubiquitously regulated probe-sets. Positive and negative regulation is denoted by a blue and red background, respectively. Whereas, probe-sets differentially regulated between shoot and root are denoted using white backgrounds.

We incorporated organ specific P_i_^starv^ treatment and recovery into one set of experiments. Briefly, we grew seedlings in P_i_ sufficient media for 20 days, followed by 10 days in P_i_ deficient (P_i_^starv^) or sufficient (P_i_^mock^) media to measure gene expression response. Plants grown in P_i_ deficient media were then transferred to P_i_ sufficient media for an additional 3 days (P_i_^replete^) for recovery. Roots and shoots were separately collected from various plant samples in 3 replicate experiments. By considering three treatments (P_i_^mock^, P_i_^starv^, and P_i_^replete^) we uncovered genes with basal expression during response and recovery, genes that initially responded to P_i_^starv^, genes that persisted (did not recover) in their initial response during recovery and genes that latently responded during the recovery phase only. These 4 states allowed us to define 9 classes of gene expression patterns (Table 1), and by comparing root and shoot responses, we were able to observe up to 81 (9^root^x9^shoot^) classes describing organ-specific or -common gene expression patterns (Figure 1i).

**Table 1:** Response-and-recovery classification scheme for loci

**Class**	**Code**	**Response**		**Recovery**		**Cartoon representation**
		p-Value*	**log**_**2**_**(FC)**	p-Value*	**log**_**2**_**(FC)**	
**Ba**sal **R**esponse	**BAR**	*no change*		*no change*		
**C**ontinuous **P**ositive **R**esponse	**CPR**	≤ 0.001	**+**	≤ 0.001	**+**	
**I**nitial **P**ositive **R**esponse	**IPR**	≤ 0.001	**+**	≤ 0.001	**-**	
**P**ersistent **P**ositive **R**esponse	**PPR**	≤ 0.001	**+**	*no change*		
**L**atent **P**ositive **R**esponse	**LPR**	*no change*		≤ 0.001	**+**	
**C**ontinuous **N**egative **R**esponse	**CNR**	≤ 0.001	**-**	≤ 0.001	**-**	
**I**nitial **N**egative **R**esponse	**INR**	≤ 0.001	**-**	≤ 0.001	**+**	
**P**ersistent **N**egative **R**esponse	**PNR**	≤ 0.001	**-**	*no change*		
**L**atent **N**egative **R**esponse	**LNR**	*no change*		≤ 0.001	**-**	

By table headings: (Class) All loci are binned into 1 of 9 response-and-recovery classes; (Code) Classes are given a 3 letter code for convenience. (Response) The *p-value* and log_2_ fold-change required by a locus’ response in order to qualify for the respective class. (Recovery) The *p-value* and log_2_ fold-change required by a locus’ recovery in order to qualify for the respective class. A “*no change*” is indicated if the *p-value* is not less than or equal to 0.001. (Cartoon Representation) A cartoon diagram indicating the general expression pattern captured by the class.

**Figure 2:**
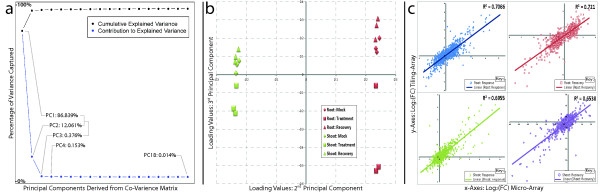
**Initial assessment of data and quality control.** Subfigure (**a**) and (**b**) summarizes all gene expression data from 18 micro-array’s by making use of a principal components analysis (PCA). (**a**) The amount of variation/information captured (blue) by each principle component (PC) is combined to obtain the total variation (black). (**b**) A plot of the two most biologically informative PCs, PC2 and PC3. Root (red) and shoot (green) organs are shown to separate along the x-axis (PC2), whereas samples for mock (diamond), treatment (square), and recovery (triangle) separate along the y-axis (PC3). (**c**) Dot plot of log_2_ fold-change values for loci captured by micro-array (x-axis) and tiling-array (y-axis) technologies. The root response (top-left, blue) and recovery (top-right, red) each have R^2^ values above 0.7, whereas the shoot response (bottom-left, green) and recovery (bottom-right, purple) each have R^2^ values above 0.65.

To increase confidence in the observed changes, we used two independent platforms to detect genome-wide expression changes. We first hybridized all cDNA samples to Affymetrix ATH1 micro-arrays*.* After having determined each chip to be of sufficient quality we continued to detect differentially regulated genes during response (P_i_^starv^/P_i_^mock^) and recovery (P_i_^replete^/P_i_^starv^) (see Additional file 2). Subsequently, we used principal component analysis (PCA) to analyze the orthogonal relationship between gene response and recovery, and between roots and shoots (Figure 2a,b). Indeed, where the 1^st^ PC likely accounted for broad variations, the 2^nd^ and 3^rd^ PCs captured true biological phenomena. For example, the 2^nd^ PC accounted for 12% of the total variation (Figure 2a) and clearly distinguished between root and shoot samples (Figure 2b-xAxis); and, the 3^rd^ PC highlighted differences between P_i_^mock^, P_i_^starv^, and P_i_^replete^ treatments (Figure 2b-yAxis). Significantly, according to the 3^rd^ PC, P_i_^mock^ and P_i_^replete^ treatments were closely related in both roots and shoots indicating recovery from P_i_^starv^ in both organs.

To increase confidence in our data we hybridized identical cDNA libraries to Affymetrix tiling-arrays 1.0R, which were processed using cisgenome software [15]. We initially evaluated data parity between the two platforms by calculating correlation of fold-change between probe-sets present on the ATH1 arrays and their corresponding probes on the tiling-arrays 1.0R. The correlation between the two platforms was above 0.8 for the root-response, root-recovery, leaf-response and leaf-recovery (Figure 2c). In addition, we confirmed transcript levels of 37 genes selected from both platforms by quantitative reverse transcriptase PCR (qRT-PCR, see Additional file 3).

### Novel P_i_-signaling patterns observed among genes involved in P_i_^starv^

#### Response-and-recovery gene expression patterns in roots and shoots

Arabidopsis roots displayed several novel responses to P_i_^starv^ not observed in previous genome-wide studies. Several previous studies have each independently identified gene sets that initially respond to [9,11,12] or recover from P_i_^starv^[10]. However, we were able to additionally identify genes that persisted in their expression during the 3-day recovery period and others that latently responded to P_i_^starv^ during recovery. Moreover, we were able to characterize these responses in an organ-specific manner. A total of 1,257 genes detected by ATH1 probe-sets (henceforth referred to as gene/s) were differentially expressed in roots during either starvation or recovery. Table 1 highlights the criteria used to sub-classify each of the 1,257 genes. A large proportion of root-responsive genes (310, ≈25%) fell into the “Initial Positive Response” class (IPR). These genes were significantly up-regulated during starvation but returned to basal levels after recovery (Figure 1e,f,g,h). One hundred and ten genes (≈8%) were negatively regulated, falling into the “Initial Negative Response” class (INR). By contrast, 47 and 50 genes continued to be up- (“Persistent Positive Response”, PPR) or down-regulated (“Persistent Negative Response”, PNR), respectively, during recovery. A significant proportion of the genes (58%) responded to recovery only after an initial priming by P_i_^starv^. We classified these latent genes into either the “Latent Positive Response” (LPR; 420 genes) or “Latent Negative Response” (LNR; 320 genes) class. Thus, the latent response of 740 genes (LPR + LNR) was of equal relevance to P_i_-signaling as genes of the initial and persistent starvation responses combined (517 genes from IPR, INR, PPR, and PNR). However, as observed in Figure 1g, latently responsive genes (red/orange) are generally regulated to a lesser extent (fold-change) than initially responsive genes (dark-green/light-green).

For shoots, using the same criteria to identify differentially expressed genes as in roots (Table 1), we classified 182 genes into response-and-recovery classes (Figure 1a,b,c,d). Of these 182 genes, 69 (≈38%) were classified as IPR whereas only 1 was determined to be INR. Briefly, we classified the remaining 112 genes as follows: 18 PPR and 2 PNR; and, 23 LPR and 69 LNR. Other than the large LPR response in roots (Figure 1h), the distribution of gene numbers between root and shoot classes were similar, with the number of latently responsive genes (92 genes) approximately equal to those of the initial and persistent responses combined (90 genes). In addition, we noted that shoot tissues initially up-regulated 48% of all responsive genes and the remainder were latently down-regulated in shoots. Hence, shoot tissues show a distinct shift in gene expression patterns, from an almost absolute initial positive response to a similarly strong latent negative response.

#### Gene expression in roots and shoots recover from P_i_^starv^ after 3 days in replete conditions

Most of the significantly expressed genes, during the initial shoot (Figure 1a-black) and root (Figure 1e-black) responses, returned to basal expression levels after a 3 day recovery in P_i_-sufficient conditions (P_i_^replete^). This is indicated by the general trend that genes had to spread across the *y = −x* axis (Figure 1c,g – dashed red line), i.e. the axis of full-recovery. We note that of the 9 response-and-recovery classes, 2 were neither observed in shoots nor roots. These two undetected classes, “Continuous Positive Response” (CPR) and “Continuous Negative Response” (CNR) described genes that responded significantly and in the same manner during both response-and-recovery phases (Table 1). The absence of these classes in our dataset, together with the general trend of our data and the expression profile of P_i_-starvation responsive noncoding RNAs *IPS1*, *miR399*, and *miR827* (see Additional file 4), confirmed that a 3-day period in P_i_ sufficient media was a suitable choice for the recovery period.

#### Co-expression versus organ specific gene expression patterns

Both roots and shoots responded to P_i_^starv^ to varying degrees by differentially regulating initial and persistently responsive genes. P_i_-signaling cascades were initiated in anticipation of P_i_^replete^ conditions, as observed by genes in the latently responsive class. The root response included 89 genes that were also differentially regulated among a total of 182 shoot responsive genes (Figure 1i-all rows and columns except the first). Yet, both organs displayed characteristic gene expression patterns. Furthermore, roots accounted for 87% of the combined P_i_-responsive genes in both organs. Consideration of the root/shoot response without the 89 ubiquitously regulated genes uncovered at least 2 tissue-specific and P_i_-sensitive regulons. The 89 co-regulated genes may be involved in systemic P_i_-signaling as 60% of these putative systemic-regulators were differentially regulated in the same manner in both organs (Figure 1i-diagonal). Thibaud et al. (2010) identified 42 systemically regulated genes of which 16 where basally regulated and 14 were considered to be regulated in both our root and shoot datasets. Of the remaining 35 genes in our study (40%), 8 and 1 were generally up- and down-regulated in roots and shoots, respectively; whereas, 26 genes displayed antagonistic behavior between the two organs.

The classification of response-and-recovery into 9 distinct classes reflected the molecular diversity in P_i_-signaling during P_i_^starv^. The independent analysis of roots and shoots has aided in the identification of systemic versus organ-specific responses. Differential regulation of systemic and organ-specific genes is indicative of function related to the P_i_^starv^ response. In the following, we present correlations between observed classes and annotations among public databases and current literature.

### The response and recovery of genes absent from the micro-array platform

A clear advantage of the 1.0R tiling-array platform over the ATH1 micro-array is the detection of transcripts derived from genomic regions not covered by the micro-array’s optimized probes. Indeed, TAIR8 has annotations for 26,956 nuclear protein-coding genes (AGI identifiers, PUBMedID: 22140109). Of these, 21,912 (81%) genes are represented by probes on the ATH1 micro-array. With this in mind we set out to characterize the transcriptional response and recovery to P_i_^starv^ of the remaining 5,044 genes undetectable by the ATH1 micro-array.

All genes with less than 3 representative probes on the 1.0R tiling-array were excluded from further analysis. For the remaining 4,730 genes, the transcript abundance for each treatment-condition was calculated using the same median-polish methodology as used in the micro-array analysis (PUBMedID: 18348734). The response of all genes (G_1-n_) in roots was assessed by normalizing according to the standard deviation (σ) and declaring a gene as differentially expressed if |G_i_/σ| > = 3. This ensured that only the most differentially expressed genes (top 1.5% in the root response) were considered to be regulated. This method was further employed to detect differential expression during root recovery, and again for the shoot response and recovery. Using this data, genes that are absent from the micro-array were classified into 1 of 9 response-and-recovery classes in a manner analogous to the classification of genes described by Table 1. This method classified 477 out of the 4,730 tiling-array-specific genes as differentially expressed (Table 2); 171 genes were root specific, 242 were shoot specific, and 64 were systemically regulated. Details on the response and recovery for all 4,730 genes can be found in supplemental data (see Additional file 5).

**Table 2:** Responsive and recovering genes absent from the ATH1 micro-array

**Identifiers**		**Root**			**Shoot**		
**AGI**	**Symbol**	Res.*	Rec.*	**Class**	Res.*	Rec.*	**Class**
		* Systemically Regulated*					
AT4G01060	*CPL3/ETC3*	4.291	−3.748	IPR	1.476	−1.505	IPR
AT5G43300	*GDPD3*	4.272	−4.045	IPR	2.121	−1.893	IPR
AT3G09922	*IPS1*	5.971	−4.591	IPR	6.572	−5.800	IPR
AT4G19038	*LCR15*	1.376	−2.223	LNR	1.143	−1.756	LNR
AT3G61172	*LCR8*	0.412	−1.981	LNR	0.518	−1.667	LNR
AT2G41240	*BHLH100*	−2.631	1.024	PNR	−3.980	1.716	INR
AT3G07005	*LCR43*	−1.692	0.274	PNR	1.687	−0.321	PPR
AT1G73607	*LCR65*	−2.178	−0.235	PNR	1.417	−1.490	IPR
AT3G56970	*ORG2*	−1.625	0.454	PNR	−4.283	1.894	INR
AT3G61177	*LCR53*	1.963	−1.140	PPR	−1.302	0.300	PNR
			* Root Specific*				
AT1G73165	*CLE1*	−1.977	1.924	INR	−0.107	−0.097	BAR
AT2G31081	*CLE4*	−2.012	2.875	INR	1.168	−0.746	BAR
AT2G31082	*CLE7*	−1.952	2.312	INR	0.111	0.121	BAR
AT5G24920	*GDU5*	−2.877	3.590	INR	0.217	0.223	BAR
AT3G06985	*LCR44*	−1.885	2.264	INR	1.002	−0.650	BAR
AT3G49570	*LSU3*	−2.308	1.684	INR	0.613	0.096	BAR
AT4G18197	*PUP7*	−1.770	1.755	INR	0.544	−0.300	BAR
AT1G54760	*AGL85*	1.941	−2.146	IPR	−0.136	−0.280	BAR
AT5G06905	*CYP712A2*	2.089	−2.979	IPR	−0.110	−0.599	BAR
AT3G30725	*GDU6*	3.627	−5.125	IPR	−0.030	−0.020	BAR
AT2G32960	*PFA-DSP2*	1.810	−1.706	IPR	1.185	−1.223	BAR
AT3G09400	*PLL3*	2.523	−2.929	IPR	0.978	−0.054	BAR
AT4G27920	*RCAR4*	2.259	−2.132	IPR	0.734	−0.431	BAR
AT1G53130	*GRI*	−0.375	−1.756	LNR	−0.056	−0.117	BAR
AT3G61182	*LCR54*	0.226	−1.759	LNR	−0.763	−0.202	BAR
AT2G14365	*LCR84*	0.519	−1.936	LNR	0.756	−0.101	BAR
AT4G10115	*SCRL20*	0.348	−2.985	LNR	−0.297	−0.485	BAR
AT4G29305	*LCR25*	−1.368	2.295	LPR	0.973	−0.521	BAR
AT4G06746	*RAP2.9*	−1.617	3.125	LPR	−0.404	0.512	BAR
AT4G23170	*EP1*	−2.487	0.662	PNR	−0.033	−0.134	BAR
AT4G09795	*LCR13*	−2.395	1.119	PNR	−0.939	0.103	BAR
AT3G23167	*LCR39*	−1.694	0.502	PNR	0.556	−1.297	BAR
AT4G18195	*PUP8*	−1.820	1.335	PNR	−0.136	−0.001	BAR
AT3G23715	*SCRL13*	−2.088	0.971	PNR	1.289	0.501	BAR
AT2G20825	*ULT2*	−1.640	0.337	PNR	0.339	0.139	BAR
AT5G45105	*ZIP8*	−2.392	0.846	PNR	0.284	−1.101	BAR
AT4G22210	*LCR85*	1.664	0.028	PPR	0.399	−0.238	BAR
AT1G60815	*RALFL7*	1.728	−0.550	PPR	0.315	0.319	BAR
			* Shoot Specific*				
AT1G47510	*AT5PTASE11*	0.182	−0.184	BAR	−1.362	1.610	INR
AT1G66145	*CLE18*	−0.570	−0.135	BAR	−1.422	1.680	INR
AT4G11485	*LCR11*	−0.969	−1.131	BAR	−1.695	1.849	INR
AT4G13890	*SHM5*	−0.177	−0.165	BAR	−1.534	1.421	INR
AT4G10767	*SCRL21*	−0.211	−0.470	BAR	1.459	−2.258	IPR
AT2G30432	*TCL1*	0.062	−0.924	BAR	1.680	−1.893	IPR
AT1G06280	*LBD2*	−0.667	−0.194	BAR	0.729	−1.385	LNR
AT4G29280	*LCR22*	−0.461	−0.171	BAR	0.485	−1.779	LNR
AT3G04430	*NAC049*	1.550	−0.809	BAR	0.731	−1.440	LNR
AT1G23147	*RALFL3*	−0.093	−0.201	BAR	1.262	−2.229	LNR
AT5G45875	*SCRL27*	0.864	0.235	BAR	0.754	−1.712	LNR
AT4G31380	*FLP1*	0.549	−0.073	BAR	−0.234	1.555	LPR
AT4G29283	*LCR21*	−1.583	−0.275	BAR	0.150	1.783	LPR
AT2G14935	*LCR40*	0.461	0.290	BAR	−0.469	1.423	LPR
AT2G12465	*LCR50*	0.500	−1.178	BAR	−0.942	1.510	LPR
AT2G02147	*LCR73*	0.599	1.466	BAR	−0.563	1.383	LPR
AT2G04425	*LCR82*	1.334	0.118	BAR	−1.264	1.380	LPR
AT4G36950	*MAPKKK21*	0.228	−0.210	BAR	−0.776	1.478	LPR
AT5G44430	*PDF1.2 C*	0.682	−1.123	BAR	−0.528	1.747	LPR
AT2G05117	*SCRL9*	−0.182	0.668	BAR	−1.005	1.595	LPR
AT5G37415	*AGL105*	0.684	−0.214	BAR	−1.931	0.608	PNR
AT2G45110	*EXPB4*	−0.018	0.286	BAR	−1.495	0.106	PNR
AT1G07900	*LBD1*	−0.174	−0.103	BAR	−1.834	0.837	PNR
AT3G43083	*LCR33*	−0.830	0.966	BAR	−1.956	0.748	PNR
AT4G39917	*LCR45*	−0.321	0.020	BAR	−1.897	0.452	PNR
AT5G14490	*NAC085*	0.099	−0.557	BAR	−1.365	0.724	PNR
AT4G11653	*RALFL29*	−0.280	−0.607	BAR	−1.900	0.762	PNR
AT1G60625	*RALFL6*	−0.668	0.726	BAR	−1.671	0.373	PNR
AT4G24230	*ACBP3*	1.295	−0.391	BAR	1.546	−1.194	PPR
AT4G29273	*LCR23*	0.128	−0.851	BAR	1.352	−0.802	PPR
AT2G19020	*RALFL10*	−1.217	0.276	BAR	1.745	−1.112	PPR
AT2G34825	*RALFL20*	0.240	0.456	BAR	1.375	−0.603	PPR
AT5G08150	*SOB5*	1.213	−1.337	BAR	1.944	−1.205	PPR

This table presents 71 of the 477 tiling-array-specific genes identified as differentially expressed during the P_i_^starv^ response and/or recovery. These 71 were chosen only for the reason that they have been annotated with a gene symbol. The remaining 406 genes do not have gene symbols and are poorly annotated. See Additional file 5 for a complete list. *: Abbreviations for response (Res.) and recovery (Rec.).

### Analysis of functionally related gene loci

Using GO-SLIM, we collected annotations and calculated *p-values* for all genes classified in both root and shoot datasets (1350 genes). The highest ranked term was, “cellular response to phosphate starvation” (Bonferroni adjusted *p-value* = 3.81 × 10^-13^). Thirteen of the 21 genes annotated by this term were identified in genes expressed in both organs and they predominantly exhibited an IPR pattern. Several additional terms such as “galactolipid biosynthetic process” and”sugar:hydrogen symporter activity” were identified as significant. As expected, these terms described genes already known to be P_i_ responsive. Therefore, we aimed to extend current knowledge by focusing on gene expression changes in the context of response-and-recovery and in the two separate organs.

#### Expression patterns of known P_i_ responsive gene loci in roots and shoots

**Figure 3:**
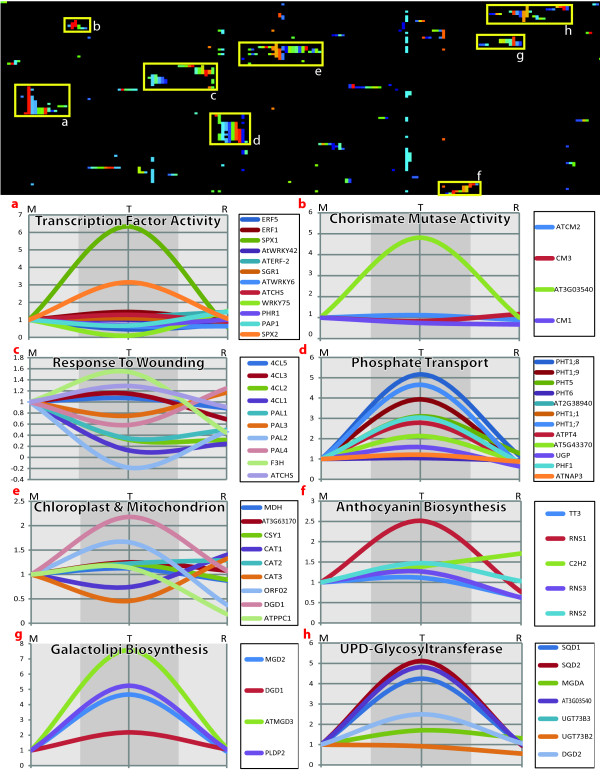
**Analysis of gene expression and function among known P**_**i**_**-responsive loci.** (Top) Clustered heatmap of known P_i_-responsive loci (rows) and GO-SLIM annotations (columns). Each color represents a different functional annotation and colors are only displayed, per row: if the rows gene is annotated by the GO-SLIM term. Selected clusters are marked from **a**-to-**h**. (Bottom, a-h) Transcription profiles of each functional cluster displaying the relative abundance of transcripts for particular known P_i_-responsive genes in mock (M), treatment (T), and recovery (R) samples.

To facilitate comparison to prior studies, we were able to identify a total of 84 genes (AGI identifiers) that have been variously described in the literature and annotated by TAIR as being P_i_-responsive (see Additional file 6). We clustered these 84 genes by their functional annotations (Figure 3-heatmap) and identified 8 partially overlapping functional clusters. Figure 3a-h shows the gene expression patterns for these known P_i_-responsive genes during both starvation and recovery in our root dataset. Generally, these 8 clusters grouped genes involved in transcription, plastid metabolism, response to wounding, P_i_-transport, anthocyanin biosynthesis, and galacto- and glyco-lipid biosynthesis. Using a Bonferroni corrected *p-value* threshold of 0.001 we classified 30% of the 84 genes as IPR in roots. However, the majority (60%) of the known loci did not significantly respond. This is perhaps not surprising because these genes were aggregated from the results of many studies, each employing a distinct protocol. Of the remaining 10%, *PEPCK2* displayed a persistent positive response to starvation whereas *AAT* a persistent negative response. Two separate sets of genes each responded in roots in a latent manner: LPR (*PAP1, CAT3, PHO1*) and LNR (*UGP, ORF02, ATPPC1, LPR1*). Similar results were obtained when we performed the same analysis on gene expression levels in shoots. Comparison of the two organs showed that 14 genes (*ATPAP1, ATPAP17, PHT1;4, PHT1;7, PHO1;H1, PHF1, PLDP2, MGD2, MGD3, SQD1, SQD2, SPX1, SPX2, and SPX3*) shared IPR patterns among both. By contrast, the majority of the remaining IPR genes (*At3g03530, DGD2, PAP6, PHT1;3, PHT1;1, PHT1;8, PHT1;9, PHT1;5*) were non-responsive in shoots.

For the following functional analyses, gene lists were converted from lists of Affymetrix probe-ids to AGI identifiers. This step was necessary to maintain the accuracy of results, as the relationship between probe-ids and AGI identifiers is many-to-many, but probe-ids to transcript abundance and AGI-ids to functional-terms are one-to-one relationships.

In contrast to the 84 known P_i_-responsive loci described above, we uncovered a more complex network of 1231 AGI gene identifiers that were also involved in root P_i_^starv^. Among these putative P_i_-responsive genes we distinguished between those involved in the initial response from those in the latent response to P_i_^starv^. Upon further examination, we found that a small subset of 93 genes were neither aptly described as initial nor latent in their response patterns. Instead, these genes persisted in their initial expression and did not return to basal levels after a 3-day recovery; this was likely due to an insufficient recovery period for this group of genes. Thus, we investigated gene expression and function of these novel root P_i_-responsive loci among 3 phases: ‘INITIAL’, ‘PERSISTENT’, and ‘LATENT’ (see Additional file 7).

#### Three gene expression phases uncover functional responses to P_i_^starv^

Considering both the response to and recovery from P_i_^starv^, we classified a gene’s expression profile into one of three phases – initial, persistent, and latent. By definition, for a gene to be classified it had to be significantly differentially expressed in either the response to and/or recovery from P_i_^starv^. Thus, each phase may include genes that were either positively or negatively regulated. In this section we focus on genes that were root P_i_-responders but not members of the group of 84 genes previously known to be P_i_-responsive. Hence, all gene counts have been adjusted to exclude known P_i_-responders. Genes that were responsive in the root’s initial response phase consisted of 292 positively (IPR) and 112 negatively (INR) regulated genes whereas those persistently responsive included 45 positively (PPR) and 48 negatively (PNR) regulated genes. Finally, the root’s latent response phase consisted of 423 positively (LPR) and 311 negatively (LNR) regulated genes.

**Figure 4:**
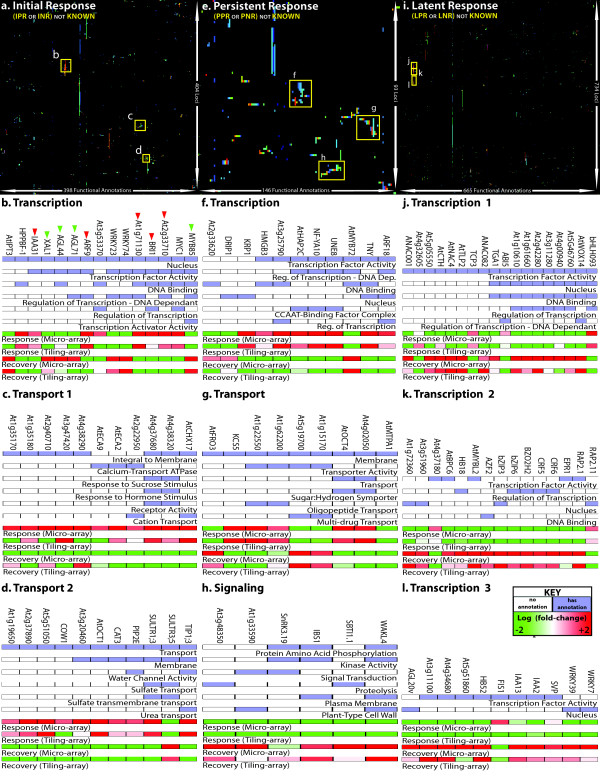
**Analysis of gene expression and function among novel P**_**i**_**-responsive loci.** (Top, a, e, i) Clustered heatmaps of novel P_i_-responsive genes (rows) and GO-SLIM annotations (columns) separately displayed for the initial (**a**), persistent (**e**), and latent (**i**) responses. (**b-d, f-h, j-l**) Summaries of selected clusters from clustered heatmaps showing annotations (blue: annotated; white: not annotated) and fold-change for response-and-recovery (red: positively regulated; green: negatively regulated) as measured by micro- and tiling-array platforms.

To ascertain function, classified genes were annotated with GO-SLIM terms by which initial, persistent, and latent gene groups were annotated with 398, 146, and 665 functional terms, respectively. tMeV software [16,17] was employed to cluster functionally similar genes (Figure 4a (initial), 4e (persistent), 4i (latent)). Using functional-clusters we determined if gene members of any one functional-cluster displayed a stronger response than those of any other. To this end, we overlaid expression data with the heatmaps produced by tMeV (Figure 4). Figure 4 displays the results for each gene expression phase by highlighting results from the initial phase in the left column (Figure 4b-d), the persistent phase in the middle column (Figure 4f-h) and the latent phase in the right column (Figure 4j-l). Three prominent functional clusters were highlighted for each phase: Genes were displayed across columns, functional annotations were shown as rows, and gene expression was indicated for both micro- and tiling-array root datasets.

Initially responsive gene clusters generally performed functions in “transcription regulation” (Figure 4b), “ionic transport” (Figure 4c), and “transmembrane transport” (Figure 4d). Among the transcription factor (TF) genes identified, *AGL44, AGL71, XAL1(AGL12), MYB85*, and *WRKY23* were significantly down-regulated in roots (Figure 4b-green arrow). The agamous-like (AGL) TFs are known to be involved with root morphological processes [18], SOC1 is known to interact with both AGL44 and XAL1 [19], and MYB85 regulates lignin biosynthesis [20,21]. On the other hand, *ARF9, BRI1, IAA31, At1g71130*, and *At2g33710* were up-regulated (Figure 4b-red arrow). Both *At1g71130* and *At2g33710* are members of the *ERF/AP2* family with *At1g71130* being involved in sugar:phosphate studies and *At2g33710* in salt-stress. Thus, among others mentioned, *At2g33710* encodes a TF candidate gene that deserves further studies with regards to its role in P_i_-signaling. The second cluster of ionic transport genes were all up-regulated in roots in response to P_i_^starv^. Three gene members of this cluster (*ECA9, ECA2, At2g22950*) encode proteins involved in calcium ion transport. *ECA9* is an auto-regulated Ca^2+^ efflux pump and the *ECA2* ATPase catalyzes Ca^2+^ efflux. Similarly, *ATCHX17* is a sodium/proton anti-porter and the gene for this protein was expressed 6.4 fold higher in P_i_^starv^ than in the control, subsequently reducing its expression level by 5 fold during recovery. The third cluster included up-regulated genes encoding transporters. Most notably, genes for the sulfate and carnitine transporters, *SULTR1;3* and *OCT1,* were up-regulated by 7 and 71.5 fold in P_i_^starv^, respectively. Indeed, *OCT1* represented the most differentially expressed gene locus in this study.

Gene loci observed as persistently responsive were generally involved in “transcription regulation” (Figure 4f) and “ionic transport” (Figure 4g). These two functions were common to those seen among the initially responsive gene loci. However, a new function was also seen: “intra-cellular signaling” (Figure 4h). Of the TF genes (Figure 4f), *MYB72* was the most differentially regulated locus in root (7 fold). This was followed by *At3g25790,* a putative MYB transcription factor (TF), which was up-regulated by 3.5 fold. The ethylene responsive TF gene, *TNY (DREB A-4)*, exhibited a PPR expression pattern. It is involved in cytokinin biosynthesis [22], and cytokinin signaling is known to cross-talk with P_i_^starv^ signaling [23]. Additionally, the *DRIP1* gene identified as a member of the PNR class, encodes an E3 ligase known to mediate ubiquitination of *TNY*’s family member *DREB2A*[24]. Finally, three CCAAT-binding TF genes were significantly up-regulated: *NF-YA3* (HAP2C, 1.8 fold), *NF-YA2* (HAP2B, 2.5 fold) and *NF-YA10* (2.4 fold). The second persistently responsive gene cluster included genes coding for membrane-associated transport proteins (Figure 4g). *MTPA1,* a zinc-ion efflux transporter, was the most differently expressed in root (down-regulated by 10.2 fold). The gene for the iron-reductase, *FRO3*, also displayed a persistent negative response of 2.8 fold. By contrast, two peptide transporter genes, *At1g62200* and *At1g22550*, identified as members of the PPR class, have been implicated in zinc hyper-accumulation. And, the gene for another carnitine transporter, *OCT4*, displayed a PPR pattern with a 2.5 fold increase in transcript levels. In contrast to *OCT1’s* IPR pattern (fold-change of 71.5), *OCT4* did not return to basal expression levels during the recovery. The third persistently responsive gene cluster contained a mix of terms related to molecular signaling (Figure 4h). All genes within this cluster were negatively regulated and did not recover after 3 days in the replete medium. The most prominent members were *WALK4* (3.4 fold down-regulated) and *CIPK22* (3.3 fold down-regulated). The former encodes a member of the membrane-bound receptor-like kinase super family whereas *CIPK22* encodes a protein known to associate with calcium-binding calcineurin B-like proteins [25]. Finally, *At1g33590* which is involved in signal transduction and the karrikin response was persistently down-regulated by 2.6 fold [26].

Among the latently responsive gene loci the most prominent expression patterns belonged to clusters implicated in transcription regulation. In total, we identified 48 TF genes that were differentially expressed in roots during the recovery from, but not the response to starvation. This number exceeded the number of TF genes identified in the initial and persistent root response by 4 and 5.3 fold, respectively. Among proteins encoded by these 48 TF genes, several TF families were prominently represented: (1) the major leucine zipper family including 5 *bZIP*, 5 *bHLH*, 2 *WRKY*, and 2 *HB* TFs; (2) the *ERF/AP2* family, with 5 TFs; (3) the zinc finger family, with 4 TFs; and (4), the *NAC* family, with 3 TFs. The remaining 22 TF genes were distributed among those encoding *MYB*, *WUS*, *IAA*, and other families. Of the first family of major leucine zippers, 14 were distributed across *bZIP*, *bHLH*, *WRKY*, and *HB* sub-families. Members of the *bZIP* family are often involved in oxidative and pathogen defense responses and are commonly linked to ABA-related pathways [27,28]. In this group, genes for *BZIP3*, *BZIP6*, *BZIP9*, and *BZIP24* were differentially regulated in root during recovery from, but not response to P_i_^starv^. The *bHLH* gene family which is generally involved in plant development, circadian rhythm, and stress [29] included genes for *BHLH093*, *MYC3*, *At1g10610*, *At1g61660*, and *At2g42280*. The WRKY family members which mediate salicylic and jasmonic acid signaling are involved in defense against pathogens and/or herbivores [30]. We found *WRKY7* and *WRKY39* to latently respond to P_i_^starv^. Finally, we identified *HD-ZIP18* and *HD-ZIP52* as two latently responsive genes. The second family of ERF/AP2 TFs are involved in acclimation stress responsive to salicylic acid, jasmonic acid, cytokinin, and ethylene [31,32]. Here, we identified 2 cytokinin response factors including CRF5 and CRF6 whose genes were up-regulated during the recovery period. Genes for 3 ethylene response factors, *RAP2.1*, *RAP2.11*, and *HRE1*, were found to express differentially during recovery. Members of the third major TF gene family encode zinc-fingers, of which we identified 4 positively regulated candidates, *ZF2, AT4G00940, ATCTH, GATA3*. The final pertinent TF genes identified were 3 members of the *NAC* family, *NAC001, NAC080, and NAC082*, which were also up-regulated in a similar manner.

The functional diversity across the latently expressed TF gene families prompted us to attempt to identify common processes among them. To this end we employed the TAIR database that provides associations of peer-reviewed articles to genomic loci described within said articles. Collating articles associated to latently expressed TF genes, we used a simple text-mining approach to group TF genes by previous research (see Additional file 8). We found 10 TF genes (*BZIP9, CRF6, MYBL2, NAC001, TGA1, ZF2, At1g61660, At2g03470, At4g32605, At4g37180)* previously identified in studies on pathogen response and cell-cycle regulation during geminivirus infection [33]. A separate set of 7 TF genes (*MDB2, TLP2, ZF2, At1g08170, At2g03470, At3g11100, At4g37180*) previously implicated in pollen germination and tube growth were latently regulated in roots [34]. Furthermore, 5 latently responsive TF genes (*BHLH093, BZIP9, HB52, RAP2.1, TLP2*) were reported in a cold-acclimation study [35]. Additional smaller groups of TF genes were identified in publications investigating topics such as “primary and secondary metabolites” [36], “post-transcriptional regulation” [37], “sucrose” [38,39], and “basal resistance to pathogens” [40]. Many TF genes were found to be shared across several studies. Together, these results suggested novel roles for these TF genes in roots during recovery from P_i_^starv^. On the other hand, they also highlighted the importance to consider which P_i_-responsive genes were P_i_-specific or non-specific. In the following section, we addressed these issues for all differentially regulated genes by comparing our results with those published by the AtGenExpress initiative.

### Interaction between P_i_-responsive genes and those responsive to various AtGenExpress treatments

**Figure 5:**
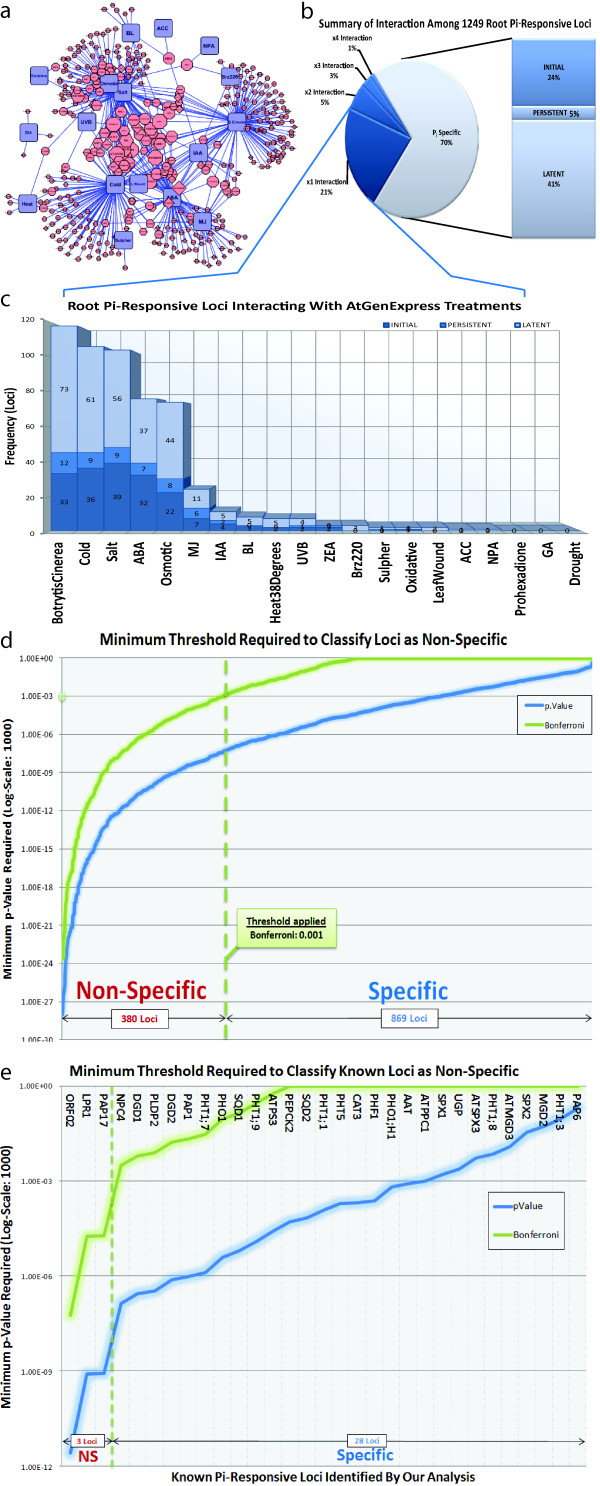
**Comparative analysis between P**_**i**_^**starv**^**data and AtGenExpress.** Subfigure (**a**) depicts a network of interactions between several types of treatment (blue squares) and genomic loci (other nodes) represented by probes on the ATH1 micro-array. Edges/lines are drawn between treatments and loci if the locus is differentially regulated in the treatment as compared to its control (see Additional file 9). (**b**) A pie chart summarizing the degree to which loci were found to interact with AtGenExpress treatments. Seventy percent of P_i_-responsive loci are found to interact with nothing else, whereas 21% interact with 1 other AtGenExpress treatment and 1% interact with 4 AtGenExpress treatments. (**c**) Stacked histogram breaking down the degree of interactivity in terms of locus counts (y-axis) between various AtGenExpress treatments (x-axis). From bottom-to-top the stacked bars indicate the contributions made by the initial, persistent, and latent responses. (**d**) A plot describing the minimum threshold required (y-axis; *p-value*: blue; corrected: green) in order to detect a locus (x-axis; ranked by y-axis) as being differentially regulated in at least 1 AtGenExpress treatment. A dotted green line indicates the default threshold used by our analysis that results in 380 loci being detected in at least 1 AtGenExpress treatment and are therefore classified as non-specific. (**c**) A plot identical to (**d**), except only a subset of loci (x-axis) that are known to be P_i_-responsive are displayed.

We defined interaction as a gene differentially regulated in at least 2 treatments. With differential regulation being determined by statistical significance (α = 0.001, using Bonferroni correction for multiplicity). To analyze possible interactions between the P_i_ response and several hormonal, environmental, and nutritive treatments, we constructed a network using the following steps: (1) We analyzed datasets from various AtGenExpress treatments (with ≥ 2 replicates) using the same methods and software as those for the analysis of our P_i_ dataset (i.e. a Bonferroni-corrected *p-value* of ≤ 0.001 versus respective control); (2) Genes differentially regulated in our root experiments were associated to AtGenExpress treatments for which they were differentially expressed; (3) We then weighted each gene-node by the number of treatments it was found to be differentially regulated and each treatment-node by the number of P_i_-responsive genes found to be differentially regulated by that treatment. In this way, the network (Figure 5a) represented the degree to which genes interact with other treatments and which treatments elicit similar regulons to those initiated by P_i_^starv^ in the roots. Thus, we described: (1) genes found to be specific to P_i_^starv^ with no interaction among the treatments studied (Figure 5b-P_i_ Specific); and (2) highly interactive genes providing evidence for co-regulation (Figure 5b).

#### Gene loci specifically regulated during P_i_^starv^ only

The following analysis was based on transcript abundance, therefore, Affymetrix probe-sets were used to represent genes and not AGI identifiers. By selecting a Bonferroni corrected *p-value* threshold of 0.001, we sorted 1249 root P_i_-responsive genes (Affymetrix probe-sets) into either P_i_-specific or non-specific bins (Figure 5b). Although there were actually 1257 root P_i_-responsive genes, 8 ambiguously mapped to AGI identifiers and were discarded from further analysis. Approximately 70% of the 1249 genes were P_i_^starv^ specific in roots, i.e. 869 genes were either initially (24%), persistently (5%), or latently (41%) responsive to P_i_^starv^ and did not interact with those affected in other AtGenExpress treatments.

The P_i_-specific genes included those already known to be P_i_-responsive and those that were novel factors of P_i_^starv^ response identified here. At a corrected *p-value* ≤ 0.001, 28 known P_i_-responsive genes tended to be exclusively regulated by P_i_^starv^. Because this type of Boolean analysis did not distinguish between genes in terms of their specificity, we ascertained the minimum threshold (*p-value*) required to reclassify a P_i_-specific gene as being non-specific (Figure 5d). The higher the *p-value* required, the greater the gene’s specificity to P_i_^starv^. Therefore, by ranking each gene according to this minimum *p-value* threshold, we could compare their relative interaction. For instance, among the known set of genes, we found *PHO1, PHO1;H1, DGD1*, and *PHF1* to be specifically regulated in response to P_i_^starv^ (Figure 5e). We also observed that *PAP6, PHT1;3,* and *MGD2* ranked higher than any other known P_i_-responsive genes and were therefore relatively more specific to P_i_^starv^ than to any other treatments tested. Indeed, *PAP6* displayed the most specificity to P_i_^starv^, requiring a notably relaxed threshold (*p-value* ≤ 0.2) in order to be classified as non-specific. Given the tendency of known P_i_-responsive genes to include multiple members of the same family (such as ion-transporters), we were able to ascertain the relative specificity of the family members (Figure 5e): (1) Among the *PHT1* P_i_-transporter family members, genes for PHT1;3 (42^nd^ most P_i_-specific), *PHT1;8* (226^th^ most P_i_-specific), *PHT1;1* (503^rd^), *PHT1;9* (637^th^), and *PHT1;7* (726^th^) were all P_i_^starv^ specific; (2) Among the lipid processing families, both *MGD2* and *MGD3* were highly ranked at 82^nd^ and 189^th^, respectively, whereas, *DGD2* and *DGD1* were less specific with ranks of 760 and 811, respectively; (3) Three members of the *SPX* gene family were highly ranked at 330 (*SPX1*), 246 (*SPX3*), and 114 (*SPX2*); finally, (4) *PHO1*, a well-studied gene, was less specific (ranked 684^th^) than its close homolog, *PHO1;H1* (ranked 394^th^).

After ranking all P_i_-responsive genes in our study (see Additional file 10), we found that several hundred P_i_-responsive genes identified here were more specific to P_i_^starv^ than many genes previously reported as being P_i_-responsive. For example, the top 100 most P_i_-specific genes included only 3 members previously described as being P_i_-responsive. These 3 were *PAP6* ranked 7^th^, *PHT1;3* ranked 42^nd^, and *MGD2* ranked 82^nd^. The remaining 97 genes responded to starvation in either an initial (49 genes), persistent (10 genes) or latent (38 genes) manner. The most P_i_-specific and initially responsive genes included *At4g19770* (encoding a putative chitinase, responding at 3.5 fold and recovering at 4.3 fold) ranked 1^st^, *At4g25160* (encoding a receptor-like cytoplasmic kinase with similarity to universal stress response protein of bacteria, responding at 7.5 fold and recovering at 16.5 fold) ranked 3^rd^ and *At1g04700* (encoding a tyrosine kinase, responding at 1.8 fold and recovering at 2.2 fold) ranked 5^th^. Genes that persistently responded to starvation during recovery include *MTPA1* (encoding a zinc transporter, responding at 10.2 fold) ranked 2^nd^, *At3g53770* (encoding a LEA3 family member, responding negatively at 3.8 fold) ranked 6^th^, and *MYB72* (encoding a TF mediating systemic resistance, responding negatively at 7.3 fold) ranked 8^th^. Finally, the top most specific latently responsive genes included *At1g62090* (encoding a pseudogene-protein kinase, recovering negatively at 1.6 fold) ranked 10^th^, *At5g66220* (encoding a chalcone-flavanone isomerase, recovering negatively at 1.6 fold) ranked 12^th^, and *At3g26130* (encoding a cellulase, recovering at 1.8 fold) ranked 13^th^.

#### P_i_-responsive genes co-regulated in multiple AtGenExpress treatments

Approximately 70% of the 1249 P_i_-responsive genes in roots identified here were P_i_-specific at a *p-value* ≤ 0.001. There was no detectable response of these genes among any AtGenExpress treatments covering stimuli ranging from hormonal and nutritional to environmental. The remaining 30% of genes (380) were not considered P_i_-specific because they differentially responded with up to 4 additional treatments found in AtGenExpress. We exploited this behavior in an attempt to elucidate: (1) treatments displaying the most similarity to P_i_^starv^ in terms of differentially regulated genes; (2) gene interaction and regulation by hormonal treatments; and (3) cohorts of co-regulated genes.

**Figure 6:**
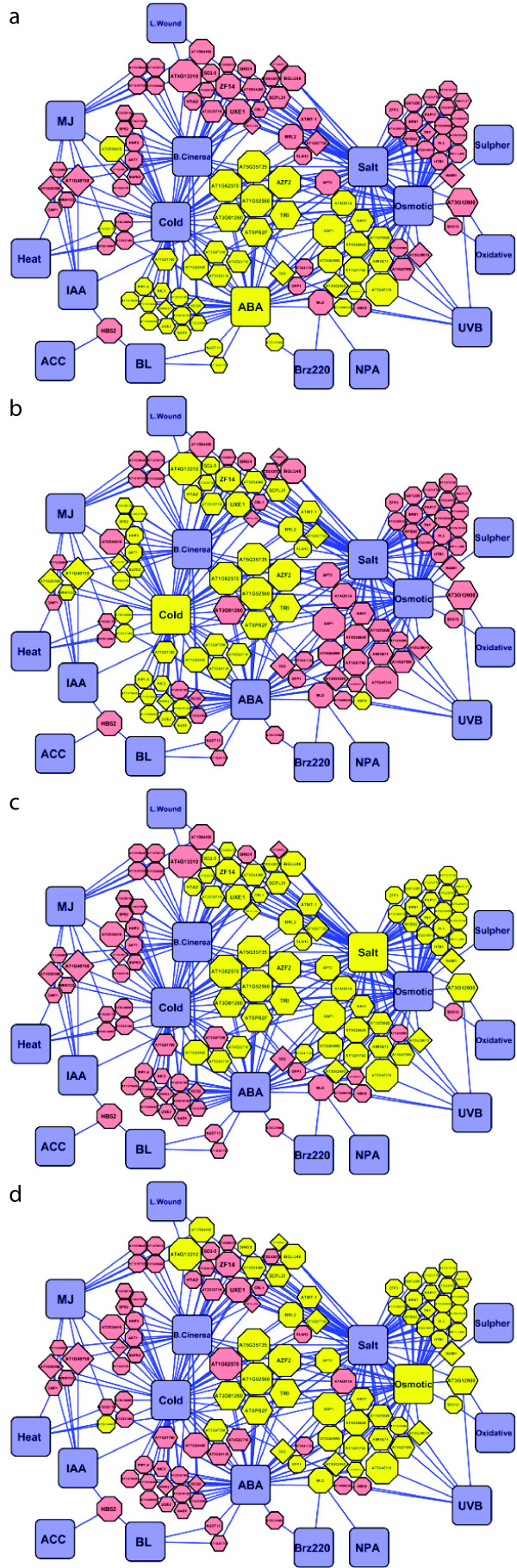
**Cross-talk between P**_**i**_^**starv**^**, ABA treatment and cold, salt, and osmotic stress.** Subfigures (**a**) through (**d**) display highlighted portions of Figure 5a. P_i_-responsive loci are highlighted (yellow) if they have additionally been found to be regulated by ABA treatment (**a**), cold stress (**b**) salt stress (**c**), and/or osmotic stress (d). The degree of overlap between highlighted regions is high indicating a large degree of cross-talk between treatments.

To determine which treatments were most similar to P_i_^starv^, we considered only those genes significantly regulated. We counted the frequency of genes differentially regulated in both P_i_^starv^ and each AtGenExpress treatment. We then weighted treatment-nodes by this frequency and ranked them (histogram, Figure 5c). Our results showed that *Botrytis cineria* infection (118 common genes) followed by cold (106), salt (104), and osmotic (74) stress treatments were most similar to P_i_^starv^. Furthermore, most genes interacting with these treatments were latently responsive in our experiments, and were mostly associated to one other non-P_i_-related treatment. We found that the maximum degree of interaction displayed by any P_i_-responsive gene was 4 AtGenExpress treatments (Figure 5b, Figure 6a-d).

Among the hormone treatments tested (ABA, ACC, BL, GA, IAA, MJ, and ZEA) ABA treatment with 76 common genes displayed the most interaction with P_i_^starv^. After which, the frequency declined sharply to MJ treatment (24 common genes), IAA treatment (11), BL treatment (8), ZEA treatment (3), ACC treatment (1) and GA treatment (0). Close inspection of those P_i_-responsive genes associated with ABA treatment uncovered many genes related to cold, salt, and osmotic treatments (Figure 6a-d). Of the 10 genes interacting with ABA, cold, salt, and osmotic treatments, only *AZF2*, *CYP706A2, SPS2F*, and *TRI* have been annotated in public databases. Not surprisingly, salt and osmotic treatments shared several responsive genes; these genes were also P_i_-responsive and included *AAP1, MYB74, and SS2*.

Overall, inspection of the interaction-network uncovered clusters of co-regulated genes. The most central cluster of genes consisted of those associated with treatments most similar to P_i_^starv^. These genes included *AZF2* (zinc finger protein, known to be regulated by ABA, salt, and desiccation), *SP2F* (putative sucrose-phosphate synthase activity), *TRI* (tropinone reductase), *At1g52560* (HSP20-like), *At1g62570* (Flavin-Monooxygenase family implicated in multiple-stress studies), *At3g01260* (Galactose-mutarotase family), and *At5g35735* (Galactose-mutarotase family). These clusters of interacting genes suggest co-regulatory mechanisms and may therefore lead to the discovery and/or elucidation of possible novel P_i_-responsive network.

## Discussion

High-throughput technologies recently applied to studying P_i_^starv^ in Arabidopsis have contributed to the overall understanding of how plant molecular systems react to P_i_-limiting stress [9-14]. However, comparing results from these studies is problematic due to different experimental designs. For example, Misson et al. (2005) [9] focused on leaf and root tissues, whereas Muller et al. (2007) [11] examined shoot tissues; Muller et al. (2007) additionally studied those genes that interact with pathways evoked during sucrose limiting stress. These studies shared the overall strategy to investigate a plant’s initial responses to P_i_^starv^. Only one high-throughput study has thus far characterized the molecular response during recovery from P_i_^starv^[10]. This work demonstrated that a subset of genes found to respond to P_i_^starv^ by prior research could also recover and thus identified high-confidence candidates sensitive to P_i_-flux. Together, these four studies have significantly increased our current understanding of P_i_^starv^ on a genome-wide scale. This report furthers the knowledge garnered by those four reports described above by combining their experimental designs under a single framework. Hence, the data described here offers insights into areas previously unreported, such as the transition between the initial response and the latent recovery phases, the detection of organ-specific differences in gene regulation and the combination of both to define the systemic plant response.

Thirty-six organ- and response-specific arrays were analyzed with the initial aims of generating a reference dataset for molecular responses to P_i_^starv^ to provide additional insights for those responses and to identify new P_i_-responsive candidate genes. To manage this dataset, a classification scheme was designed to broadly categorize novel response-and-recovery gene expression patterns. Using this scheme a unique and comparably large molecular response was identified in roots relative to shoots. We analyzed the interaction of P_i_-responsive genes with several hormonal, environmental, and nutritive treatments. This exercise defined non-specific P_i_^starv^ genes differentially regulated across multiple stimuli; in particular, stimuli such as cold, osmotic, and salt stress. Furthermore, the hormone ABA appeared to regulate substantially more P_i_-sensitive genes than other hormone treatments. This supports the premise that of the hormone regulators ABA is likely a key player during P_i_-flux. Significantly, the majority of genes known to respond to P_i_^starv^ were observed to be regulated by several different treatments. Thus, this known gene-set that includes many well described P_i_^starv^-sensitive genes such as *PHO1* is indeed not P_i_^starv^-specific. To place this into context we note that many genes not previously identified as P_i_-responsive, but identified as responsive here exhibit a lesser degree of cross-talk and hence occupy roles that are relatively more P_i_^starv^-specific. This insight provides opportunity for both generating and addressing additional questions regarding the regulation of molecular networks during nutrient stress. Given the nature of genome-wide technologies employed here much depends on the wealth of knowledge and annotations generated by prior researchers studying several genes using non-high-throughput methods. Therefore, a thorough literature and database survey was conducted for both those genes known to respond to P_i_^starv^ and those additional genes observed during the course of this study. Thus, we were able to report on all results in the context of novel findings by examination of candidate genes and their corresponding gene expression patterns during the response and recovery.

### Comparisons among different studies reveal the need to establish a reference dataset

Although previous experiments by various groups have, in aggregate, reported a total of 84 P_i_-responsive genes, our work here has observed 40% of these genes being responsive to starvation and recovery treatment. As this appeared to be a small proportion we addressed this issue by analyzing the benchmark set by previous genome-wide experiments: Misson et al. (2005) [9], Morcuende et al. (2006) [10], Muller et al. (2007) [11], and Nilsson et al. (2010) [12] each identified 14%, 33%, 3%, and 8% of the 84 significantly responsive genes, respectively. Therefore, due to the lack of overlapping results, it appears that the core set of known P_i_-responsive genes is highly variable and most likely dependent on the experimental approach. This between-study variation highlights the need to standardize experimental designs and to ascertain the cause behind the regulation of core P_i_-responsive genes.

Among 33 of the known 84 P_i_-responsive genes identified, the majority (75%) were found to be initially responsive and their expression returned to basal levels during recovery. Only 24% of the genes were latently responsive and the remaining 1% was persistently responsive. We propose that the bias towards the initial response is due to prior research almost exclusively focusing on the starvation response without considering recovery. Thus, known P_i_-responsive genes tend to be initially responsive. Only a few studies have examined recovery from starvation which is reflected by the lower proportion of known genes identified as being latently expressed.

### Expression profiles of P_i_-starvation responsive genes absent from the ATH1 micro-array

Even though the ATH1 micro-array lacks genome-wide coverage it has been widely used for transcriptome studies. Whereas the 1.0R tiling-array is not comprehensive it certainly interrogates more of the Arabidopsis genome than ATH1 and therefore has a distinct advantage. However, the public literature surrounding array-based studies is dominantly micro-array based. Analyses and methods using the micro-array architecture are better described and the genes represented by the ATH1 chip are, in general, better annotated. Therefore, given the goal of this study to better characterize the molecular response and recovery to P_i_^starv^, the micro-array platform was chosen as the primary means to analyze gene expression. Nevertheless, our acquisition of tiling-array data in parallel serves to both corroborate micro-array results as well as to investigate the response and recovery of those genetic elements not represented by ATH1 probes. We have determined the response and recovery profiles for all 5,044 nuclear protein coding genes absent from the ATH1 micro-array (see Additional file 5) and detected 477 genes that may play a role during and post P_i_^starv^ in roots and shoots (Table 2). Among those 477 genes we identified *IPS1* (now known as non-protein coding), *GDPD3* (a membrane-remodeling factor) and *CPL3* (a MYB TF), for which we describe their biology and response to P_i_^starv^ below.

*IPS1* is a P_i_^starv^-inducible non-coding RNA whose transcript level is regulated by a myb transcription factor, *PHR1*[41]. By a mechanism known as target-mimicry, *IPS1* is resistant against the activity of RISC-loaded miRNA399 due to its partial sequence complementarity to the mature miR399. This property enables *IPS1* the ability to quench the miR399 signal without being cleaved by the latter [42]. RT-PCR showed that the *IPS1* transcript level was inversely correlated to P_i_ availability in hydroponic media (see Additional file 4). This was also shown by tiling-array analysis of roots and shoots for which the responses (Log_2_(P_i_^starv^/P_i_^mock^)) were 6 and 6.5, respectively. The *IPS1* expression profile was the most differentially regulated profile among the 5,044 tiling-array-specific genes examined. Given the established role of *IPS1* during P_i_^starv^, this gene represents an excellent positive control for the remaining 476 genes identified by the tiling-array platform alone.

Unlike *IPS1*, the glycerophosphodiester phosphodiesterase (GDPD) gene family member, *GDPD3*, was not implicated in P_i_ homeostasis until 2011 [43]. Our tiling-array data corroborated this result as *GDPD3* (*At5G43300*) exhibited a 19 fold increase in signal intensity during the P_i_^starv^ response in roots. The function of the GDPD family has been implicated in P_i_-recycling through a membrane remodeling process that is sensitive to P_i_-flux. Together with the vacuole, phospholipid bilayers are a major reservoir of internal cellular P_i_. Membrane remodeling is a physiological response of plant cells to supply P_i_ on demand. This method of P_i_-recycling replaces phospholipids in internal cellular membranes with alternate forms such as galacto- and sulfo-lipids [44,45]. GDPD hydrolyzes glycerolphosphodiesters originating from phospholipids and produces a glycerol-3-phosphate (G-3-P) and a corresponding alcohol. The G-3-P byproduct is further degraded by acid phosphatases resulting in P_i_ accumulation [43]. This remodeling mechanism is one example of a plant-wide response to P_i_-limitation at a micro-scale, in addition to responses at the macro scale, e.g. by remodeling root architecture.

Plasticity of root architecture is related to the availability of soil nutrients under fluctuating environmental conditions. Under P_i_-limiting conditions, root architecture changes by inhibiting primary root growth and promoting increased density of both lateral roots and root hairs [46]. Cell fate of epidermal trichoblast and atrichoblast is specified by several negative cell and non-cell autonomous regulators *GL2*, *CPC*, *TRY*, and *ETC*s [46]. On the other hand, *CAPRICE LIKE MYB3* (*CPL3*) has been characterized as a positive regulator for root hair patterning [47]. Since *CPL3* is not represented by ATH1 probes and hence unlikely to be described in previous studies of P_i_ starvation, we were interested to gauge this gene’s response and recovery from our tiling-array data. Indeed, *CPL3* was initially up-regulated by 19.6 fold in roots and returned to basal levels during recovery (IPR). Whilst less remarkable the shoot response also displayed an IPR pattern of gene expression exhibiting an initial fold-change of 2.8. Therefore, the P_i_^starv^ response may involve *CPL3* to govern root hair patterning. Our identification of *CPL3*, *GDPD3*, *IPS1*, and the other 474 genes as being P_i_^starv^-responsive exemplifies the utility of tiling-arrays in P_i_^starv^ studies with regards to discovering new candidates for future research.

### A novel root response suggests roles for energy metabolism and ionic transport

Previous P_i_^starv^ research has not yet addressed root responses from the perspective of genome-wide studies. Whereas whole seedling experiments have undoubtedly captured a part of the root’s responses, the results are likely skewed towards shoot responses as we have found that in 2 week old seedlings the average root to shoot RNA-abundance ratio is 1:9 (data not shown). Here, we found that the number of responsive genes in roots was 6.9 fold higher than those in shoots. Additionally, the distribution of genes in terms of fold-change in their response-and-recovery highlights greater responses to P_i_^starv^ in roots than shoots (Figure 1). Furthermore, only 7% of P_i_-responsive genes in roots were significantly altered in shoots. These 3 observations emphasize the importance of a global analysis of the root-specific response which hitherto has been neglected. To better understand the root response, we divided all root responsive genes into 1 of 3 categories (initial, persistent, or latent), and selected 3 prominent (in terms of fold-change) clusters of functionally related genes per category. Among genes in the initially responsive category, the most prominent clusters were involved in ion and trans-membrane transport, specifically calcium and sodium ion transport (Figure 4c,d). Whereas, the third prominent cluster grouped genes functioning in transcription regulation (Figure 4b).

We found lipid metabolism to play a role in the initial and persistent response. *OCT1* was co-identified with several membrane transporters in the initial response. *OCT1* exhibited the most fold-change of any gene observed before returning to basal levels during recovery. Similarly, *OCT4* was identified in the persistent category and while it did not recover it also did not display as dramatic a fold-change as *OCT1*. *OCT1* and *OCT4* encode carnitine transporters involved in mitochondrial fatty acid metabolism. Arabidopsis mutants deficient in *OCT1* and transgenic plants overexpressing *35 S::OCT1* have been shown to promote and suppress lateral root hair development, respectively [48]. Paradoxically, *OCT1* is up-regulated 71-fold during P_i_^starv^, but lateral root hair development is positively regulated during P_i_-limitation. Thus, OCT1 appears to be uncoupled from root hair development during P_i_^starv^ and may hypothetically play a role in P_i_^starv^-induced lipid/energy metabolism.

### The root up-regulates transcription factors as the system tends toward recovery

Regulation of *AGL* expression occurred during the initial response but regulation of the *NF-YA* family members persisted after the recovery period, although the reason for this difference is presently unknown. Nevertheless, our results provide a shortlist of additional TF candidates for future research. Considering both the number of additional TF genes displaying initial and persistent responses, it is likely that the P_i_^starv^ response is regulated by more than just the two TFs (*PHR1*, *PHL1*) identified to date [13,49] as previously corroborated [12,50]. Indeed, we observed a marked increase in the number of differently regulated TF genes (48 TFs) during the latent response, which increased by 4 and 5.3 fold from the number of TF genes observed during the initial and persistent responses, respectively. These results suggest that significant regulatory changes occurred latently during the period of recovery from P_i_^starv^. Using a text-mining approach we have uncovered some P_i_-responsive TF genes to be involved in viral infection, cold acclimation and even pollen tube growth (PTG). Interaction of P_i_-responsive TF genes with genes involved in viral defense and cold acclimation can be explained if a general stress response underlies all three cases. In addition, root hair extension is promoted during P_i_^starv^ and is known to share many common features with pollen tube growth (PTG).

### Expression of IPT3, ARF9, and MYB85 may alter root-morphology during P_i_^starv^

During P_i_^starv^ the root architecture is significantly altered through both negative and positive regulation of primary and lateral root (LR) development. Root development processes are known to be down-regulated by cytokinin (CK) at high concentrations [51]. Therefore, P_i_^starv^ may be expected to reduce endogenous CK concentrations to release LR developmental pathways. Although CK concentrations were not directly measured in our experiments, we found a significant down regulation of *IPT3* in roots suggesting an additional P_i_^starv^-specific role for this gene in root CK biosynthesis.

Our results show an increased expression of *ARF9* in root tissues, although this gene has not yet been reported to play a role in root architecture nor in the P_i_^starv^ response. However, *ARF9* is known to be up-regulated by high auxin concentrations similarly to its family members *ARF7* and *ARF19,* which promote LR formation in initiating cells accumulating auxin [52]. This shows that *ARF9* does have a role in P_i_^starv^ and suggests that the role may be in morphological changes of root architecture.

Development of LR first entails the removal of structural scaffolds within cell walls, a process critical for cell expansion and proper root hair development. This process requires both the down-regulation of positive regulators (*MYB69* and *MYB85*) as well as an up-regulation of negative regulators. We found that *MYB69* and canonical upstream regulatory genes (*SIZ1, SND1, NST1, and VND6*) were all basally regulated during P_i_^starv^ in roots. Yet, expression of *MYB85* (required for proper lignin deposition) was significantly reduced. This selective down-regulation of *MYB85* suggests attenuation of lignin deposition, resulting in a favorable environment for initiation of root hairs under P_i_^starv^.

### Cross-talk among P_i_^starv^ candidates uncovers interaction with plant hormones

To observe possible cross-talk between P_i_^starv^ and hormonal responses we investigated interaction between our and relevant AtGenExpress data-sets. All AtGenExpress datasets were re-analyzed in the same manner as performed on our own. P_i_-responsive genes were considered to be specific to P_i_^starv^ if they were not differentially regulated in any treatment regime. Hence, by determining the minimum *p-value* threshold required for each gene to be categorized as non-specific we were able to rank each P_i_-responsive gene and determine its relative specificity. Using this method we showed that *MYB72* is highly P_i_-specific when compared to any other gene. This measure of relative specificity allowed us to determine which members of gene families play a primary role and which are functionally redundant in the P_i_-response. Although both *MYB72* and MYB*74* are significantly regulated the former gene is ranked 8^th^ for P_i_-specificity whereas the latter 1238^th^. By contrast, all the 3 cytochrome P450 genes (*CYP94D1*, ranked 13^th^; *CYP735A1*, ranked 24^th^; *CYP76G1*, ranked 27^th^) were highly specific to P_i_^starv^. This result suggests that they are not redundant and execute distinct functions with little overlap. We note that this analysis is heavily dependent on the number or breadth of treatments tested. To interpret highly P_i_-specific genes, we have assumed the treatments selected from AtGenExpress to be an adequate sampling of biological stimuli.

In addition to specificity, we have also attempted to measure gene interaction. Highly specific genes may lose their specificity by inclusion of additional treatments to the study. On the other hand, the less specific a gene, the more interactive it becomes and the less likely it is to change when additional treatments are included in the study. Also, highly interactive P_i_-responsive genes offer clues to P_i_-signaling in terms of co-regulation. For instance, we have determined groups of co-regulated P_i_-responsive genes that are influenced by one or more of the following treatments: ABA, cold, salt, drought, and *B. cinerea* infection. Indeed, these 5 treatments give the greatest degree of interactivity with P_i_^starv^. When compared to ABA treatment other hormone treatments such as ACC, IAA, MJ, BL, and ZEA elicit the expression of at most less than 32% of the number of P_i_-responsive genes.

## Conclusion

This study presents a genome-wide description of Arabidopsis’ response and recovery to P_i_^starv^ for both roots and shoots. Utilizing two different technological platforms to determine transcriptome changes we found that the majority of known P_i_-responsive genes were initially responsive. Whilst our analysis mainly focused on genes represented by probes on both micro- and tiling-array platforms we also identified 477 tiling-array-specific genes as being regulated by P_i_^starv^. One such gene was *IPS1* a non-coding gene important for P_i_ homeostasis that exhibited an initial fold-change in roots and shoots of 63 and 95, respectively. For research areas such as plant P_i_ starvation, where transcriptomics studies have relied heavily on micro-array data, these results highlight the utility of true genome-wide studies in the detection of coding as well as non-coding transcript levels. All together, our results show a more varied response-and-recovery molecular phenotype than hitherto recognized in the literature. This study, which presents an initial investigation into the functional aspect of these P_i_-responsive genes, has uncovered a progression of differentially regulated functional classes: from initially responsive ion-transporters and persistent cellular signaling genes to latently responsive transcriptional regulators. We hypothesize that initially responsive genes identified in this study function in immediate survival to P_i_ limiting stress – transporting P_i_ from source-to-sink whilst maintaining electrochemical gradients as indicated by the initial regulation of P_i_ and metal-ion transporters. This hypothesis is extended to include persistently and latently responsive genes whereby we surmise that persistently responsive genes participate in the transition between survival and recovery. This transition is evidenced by the increase in differential regulation of persistently responsive genes involved in cellular signaling to those latent genes coding for transcriptional regulators. Moreover, the extent of transcriptional regulators elicited by P_i_^starv^ suggests the presence of regulons separate from that of the *PHR1* regulon. Whether or not these regulons are P_i_^starv^-specific remains an open question. However, analysis of cross-talk using data generated from this study and from AtGenExpress revealed that many novel P_i_-responsive genes identified here appear to be more specific to P_i_^starv^ than previously identified P_i_-responsive regulons. Interestingly, *PHO1;H1* – a close homolog to *PHO1* with functional redundancy in P_i_-signaling – showed less cross-talk with other treatments than *PHO1*, in roots. This suggests that even though *PHO1* traditionally displays a greater response to P_i_^starv^ than *PHO1;H1*, the *PHR1*-mediated *PHO1;H1* response may be more specific to P_i_^starv^ than the *PHO1* pathway (*PHR1*-independent). By applying the same reasoning to other well-known P_i_ responsive families we observed that: 1) genes involved in the biosynthesis of galactolipids (phosolipid alternatives) in non-photosynthetic tissues – *MGD2*, *MGD3*, and *DGD2* – were highly specific to P_i_ limiting stress; 2) P_i_ transporters *PHT1;1*, *PHT1;3* and *PHT1;7–9* (regulated by *PHR1*) were specific to P_i_^starv^ whereas their other family members were not. Also, *PHT1;1* is regarded as one of the most responsive P_i_ transporters to P_i_ limitation suggesting that *PHT1;1* is the primary acting member of the *PHT1* family in the starvation response; 3) 3 of the 4 members in the *SPX* family are P_i_^starv^ specific (*SPX1-3*). Interestingly, *SPX1-3* are under *PHR1* control. Indeed, for each of the known P_i_ responsive gene families mentioned, the members that we observed to be most P_i_^starv^-specific – *PHO1;H1*, *PHT1;7–9*, and *SPX1-3* – have also been reported to be regulated by the TF *PHR1*. Finally, a large degree of cross-talk among clusters of co-regulated genes implicated ABA as the likely hormone mediator responsible for regulating common stress-responsive pathways; in particular cold, salt, and osmotic stress. In the future we will apply this approach to the analysis of additional treatments with the view of building a comprehensive stress response-and-recovery database.

## Methods

### Plant material and growth conditions

Non-sterile WT (Col-0) seeds were sowed on rock wool (Grodan Inc.) pre-soaked in basal MGRL hydroponic media^24^ and cold-treated at 4°C for 3 days. Plants were grown at 22°C under 150 μmol m^-2^ S^-1^ fluence rate and a 12 hr:12 hr light:dark cycle. To minimize environmental variation, plants were rotated in growth chambers once a day. In phosphate depleted media, 1.75 mM MES (pH 5.8) was used instead of 1.75 mM sodium phosphate (pH 5.8). All hydroponic media were aerated to maximize air supply during hydroponic culture. All plants were grown in basal MGRL hydroponic media for 20 days before being transferred to a phosphate-deficient medium. During the 10-day treatment, the phosphate-deficient medium was replaced every three days. For recovery experiments, plants previously grown in phosphate-deficient medium for 10 days were transferred to full-strength MGRL medium for an additional 3 days. Samples were collected on the 10th and 13th (10 + 3) day. Phosphate-starved shoot and root samples were collected separately at the indicated time point. Furthermore, all experiment procedures such as media replacement and sample collection were performed in the middle of day in order to minimize possible circadian effect. In parallel, control plants were grown in a full-strength MGRL media before sample collection on the 10th day after the initial 20-day growth period.

### Preparation of genechip and tiling-arrays

Total RNAs extracted by RNA easy extraction kit (Qiagen) were used for Arabidopsis Genechip ATH1 and tiling arrays 1.0R (Affymetrix). Samples included three biological replicates each for mock-treatment leaves (ML), mock-treatment roots (MR), P_i_-starvation-leaves (TL), P_i_-starvation-roots (TR), P_i_-starvation-recovery leaves (RL), and P_i_-starvation-recovery roots (RR). Probes of all 36 samples were synthesized as previously described^25,26^. The efficiency of biotin labeling was confirmed by Gel-shift assay with NeutrAvidin (Pierce) as described in Genechip Whole transcript (WT) double-stranded target assay manual (Affymetrix). Hybridization and scanning of all arrays were done at the Genomics Resource Center in the Rockefeller University following the manufacturer’s instructions (Affymetrix).

### Detection of phosphate responsive genes

Semi-quantitive RT-PCR and real-time PCR were performed to confirm expression changes of selective genes uncovered by gene/tiling arrays. Typically, 1 μg total RNA prepared by Qiagen RNA column extraction including DNase treatment (Qiagen) was used as a template for reverse transcription (Superscript III RT-PCR, Invitrogen). First strand cDNA was synthesized with an oligo dT primer or with an strand-specific primer. In parallel, *actin* transcript was used as an internal loading control and same amount of RNA without reverse transcription as a negative control. 100 ng of single strand cDNA were used to quantify expression by real-time PCR (Bio-Rad CFX96). Each ΔC(t) value and relative expression of phosphate-responsive genes was determined by Bio-Rad CFX manager program (Bio-Rad).

### Description of gene lists

Below is a description of the pertinent gene lists mentioned in this work.

General statistics (based on transcript levels):

1. 1.1257 Affymetrix probe-sets differentially expressed in roots.

2. 182 Affymetrix probe-sets differentially expressed in shoots.

3. 89 Affymetrix probe-sets commonly regulated in roots and shoot.

4. 1350 Affymetrix probe-sets identified in total (root + shoot - common).

Functional analysis (based on one-to-one relationships for terms and AGI identifiers):

5. 84 AGI identifiers previously known as Pi responsive.

6. 1231 AGI identifiers in root response, excluding the 84 previously identified.

Cross-talk analysis (based on transcript levels):

7. 1249 Affymetrix probe-sets differentially expressed in roots.

a. a.1257 minus 8 Affymetrix probe-sets which were ambiguously mapped to AGI identifiers and discarded from further analysis.

### Bioinformatics

The accession code, GSE34004, may be used to access the micro- and tiling-array data from the Gene Expression Omnibus (GEO). The computational portions of the Materials and Methods are described in Additional file 9. This file includes descriptions of the: *analysis pipeline; experimental design; quality control; normalization; gene classification; analysis of functional annotations; and analysis of P*_*i*_^*starv*^*specificity and cross-talk.*

## Abbreviations

P_i_: phosphate; P_i_^starv^: P_i_ starvation; P_i_^mock^: mock treatment; P_i_^replete^: P_i_ replete; PC: principal components; BAR: Basal Response; IPR: Initial Positive Response; INR: Initial Negative Response; PPR: Persistent Positive Response; PNR: Persistent Negative Response; LPR: Latent Positive Response; LNR: Latent Negative Response; CPR: Continuous Positive Response; CNR: Continuous Negative Response; RR: Root Response; SR: Shoot Response.

## Competing interests

The authors declare that they have no competing interests.

## Author’s contributions

The experimental design was conceived by JW, TK, MH, and NHC and all experiments were performed by JW except the computational analysis. Data was analyzed by CRM with assistance from JL, HW, XJW, and VBB. This paper was written by JW, CRM, VBB, and NHC. All authors read and approved the final manuscript.

## Supplementary Material

Additional file 1 Inter-study comparison, is a figure detailing a comparison of genes identified by various research groups to be regulated by phosphate flux. Click here for file

Additional file 2 Response and recovery (eBayes), contains statistical data generated from the ebayes function in the Limma package (R, statistical environment) for the response and recovery in both roots and shoots. Click here for file

Additional file 3 qRT-PCR, is a figure displaying qRT-PCR results for 37 loci from the top differentially expressed genes known to respond to phosphate-starvation. Click here for file

Additional file 4 **P**_**i**_** marker genes, RT-PCR of P**_i_**-starvation responsive non-coding RNAs IPS, miR399, and miR827 at 10-days P**_**i**_**-replete and -starved, and 3-days recovery.** Click here for file

Additional file 5 Expression profiles for 5,044 genes undetectable by the ATH1 micro-array platform, a table describing the response and recovery of said 5,044 genes to Pi starvation in both root and shoot. Click here for file

Additional file 6 Literature survey references, additional references used in the determination of the 84 known Pi-responsive gene-set. Click here for file

Additional file 7 Gene response and recovery class list, categorizes genes into 1 of 9 response-and-recovery classes for roots and shoots, separately. Click here for file

Additional file 8 Textmining data, is a table providing the data we collected and used to mine the text resources annotated by TAIR for each locus. Click here for file

Additional file 9 Materials and methods for Bioinformatics, the computational portion of the materials and methods section. Click here for file

Additional file 10 **P**_**i**_**-specifity rankings, is a table summarizing results for loci that we were able to assign a ranking of phosphate-specificity.** Click here for file
