# Supplementary material for: The response and recovery of the Arabidopsis thaliana transcriptome to phosphate starvation
Source: BMC Plant Biol. 2012 May 3;12:62. doi: 10.1186/1471-2229-12-62 (PMC3520718; doi:10.1186/1471-2229-12-62)
Supplement: Additional file 1 — Inter-study comparison, is a figure detailing a comparison of genes identified by various research groups to be regulated by phosphate flux. [file 1471-2229-12-62-S1.pdf]

Additional File 01: Comparison between published results demonstrating variability.

This complex figure is broken-up into 4 parts: 1/ VENN model (top-left); 2/ Summary overlap bar-chart (top-right); 3/ Detailed overlap table (middle); and 4/ Heatmap of studies (rows) and identified Pi-responsive genes (columns) (bottom).

1/ VENN model: This describes how our Pi-responsive gene-set (**W&M** – **W**oo and **M**acPherson) was compared to those of several others (multiple **C**omparison sets – "**C**"). These **C** gene-sets are represented by a blue circle. The **W&M** gene-set is separated into **res**ponse (red) and **rec**overy (green) and denoted by "**Res**" and "**Rec**", respectively. Thus, each gene in the **C** gene-set will fall into 1 of 7 categories (**i-vii**), depending on whether or not the gene has been identified as Pi-responsive by both studies (**W&M** and **C**). All genes in the comparison are represented by the "**o**" character, regardless of which study identified them. Genes unique to the **C** gene-set will fall into the "**i**" category, whereas those unique to **W&M** will fall into the "**iii**","**iv**", and "**v**" categories. Genes identified by both **W&M** and **C** will fall into one of the "**ii**", "**vii**", and "**vi**" categories.

2/ Summary overlap bar-chart: This bar-chart is separated into four sections (2-rows; 2-columns) and summarizes gene counts for 5 **C** gene-sets (color-coded), namely: Muller at al. (2007) in dark blue, Muller et al. (2007|sugar) in red, Misson et al. (2005) in green, Nilsson et al. (2010) in purple, and Morcuende et al. (2007) in light blue. The left column displays gene counts for the total number of genes in each **C** gene-set, the right column displays gene counts that are unique to the C gene-set (i.e. present in category "**I**" in the VENN model). The top and bottom row separate the data according to comparisons against **W&M**'s shoot and root gene-sets. From this figure it is evident that the gene-counts on the left are very similar to those on the right, and therefore there is very little overlap between **W&M**'s gene-sets and those contributed by other authors.

3/ Detailed overlap table: This table details the gene counts for each category (**o**, **i-vii**; described under "1/ VENN model"), for each C gene-set. **W&M** data is split into root and shoot and then compared to gene-sets identified by other authors. Any pertinent information regarding the authors gene-sets is described under the "Annotation" column.

4/ Heatmap of studies and identified Pi-responsive genes: W&M (reds, and green) and comparison gene-sets (black, blues, purple, and orange) are all color coded and arranged as rows. Columns represent all Pi-responsive genes (collected as the union of all gene-sets). There was not enough space to show all Pi-responsive genes, and so they were divided into 3 separate heatmaps. Red portions of the heatmap denote that a particular gene-set has identified the respective gene/s as Pi-responsive; black denotes the opposite. Thus this figure shows at a glance the degree of variation between gene-sets. Sub-figures 2/ and 3/ show that **W&M** data does not match well with other authors. But, this heatmap shows that no gene-set matches well with any other.

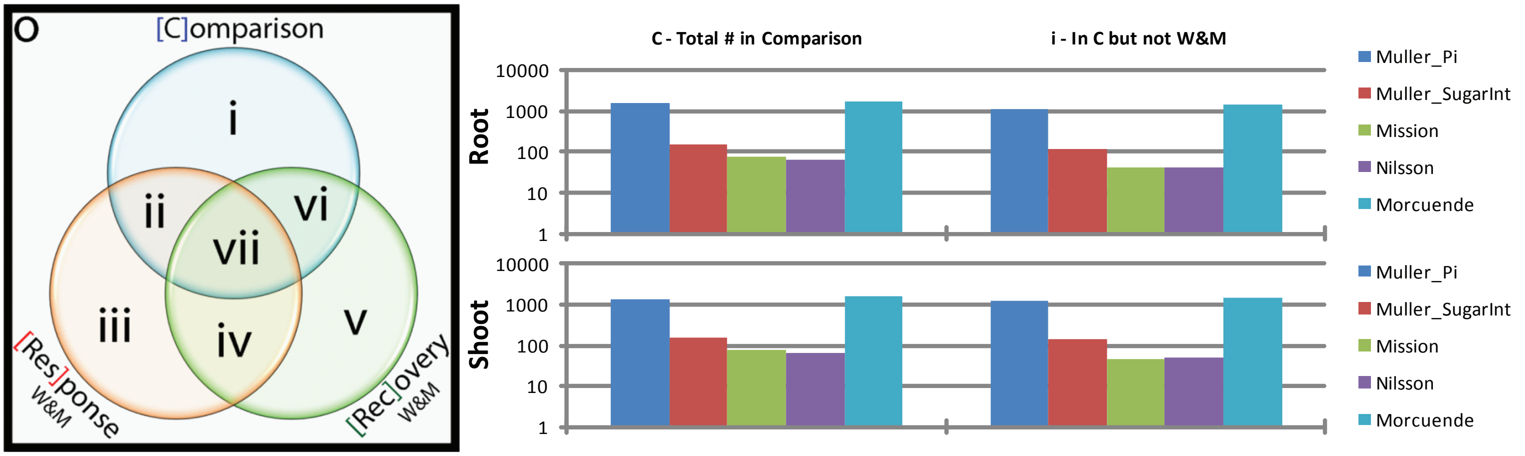

W&M Data	Compared to:		VENN Diagram Counts									
**Annotation**	**Author**	**Annotation**	**o**	**Res|Rec|C**	**C**	**i**	**ii**	**iii**	**iv**	**v**	**vi**	**vii**
**Root**	Muller_Pi	Response; Shoot	21191	2418	1586	1131	19	78	320	701	53	116
	Muller_SugarInt	Sugar Interaction; Shoot	22204	1405	149	118	1	96	415	745	9	21
	Mission	Response; Shoot + Root	22279	1330	76	43	1	96	406	752	2	30
	Nilsson	Response; Shoot + Root	22279	1330	64	43	5	92	421	753	1	15
	Morcuende	Recovery; Shoot + Root	20952	2657	1619	1370	17	80	277	681	73	159
**Shoot**	Muller_Pi	Response; Shoot	22186	1423	1319	1237	7	14	23	67	26	49
	Muller_SugarInt	Sugar Interaction; Shoot	23280	329	149	143	1	20	67	93	0	5
	Mission	Response; Shoot + Root	23379	230	76	44	0	21	46	87	6	26
	Nilsson	Response; Shoot + Root	23373	236	64	50	0	21	60	91	2	12
	Morcuende	Recovery; Shoot + Root	21910	1699	1619	1513	12	9	8	63	30	64

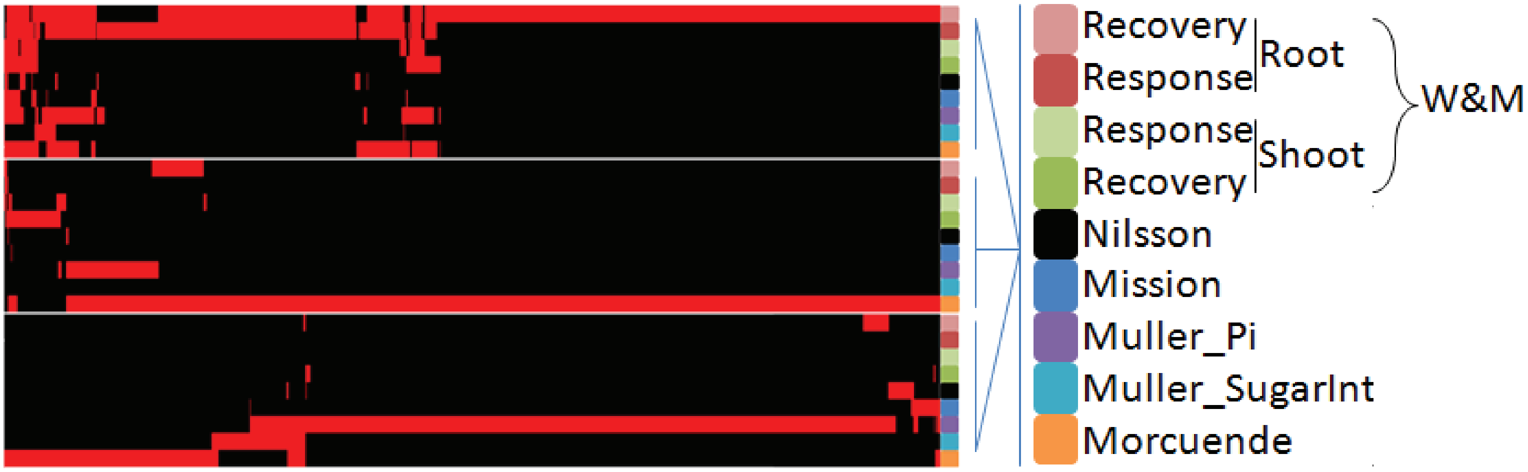

Supp. Figure 2: Comparison between published results demonstrating high variability
