# Supplementary material for: The response and recovery of the Arabidopsis thaliana transcriptome to phosphate starvation
Source: BMC Plant Biol. 2012 May 3;12:62. doi: 10.1186/1471-2229-12-62 (PMC3520718; doi:10.1186/1471-2229-12-62)
Supplement: Additional file 3 — qRT-PCR, is a figure displaying qRT-PCR results for 37 loci from the top differentially expressed genes known to respond to phosphate-starvation. [file 1471-2229-12-62-S3.pdf]

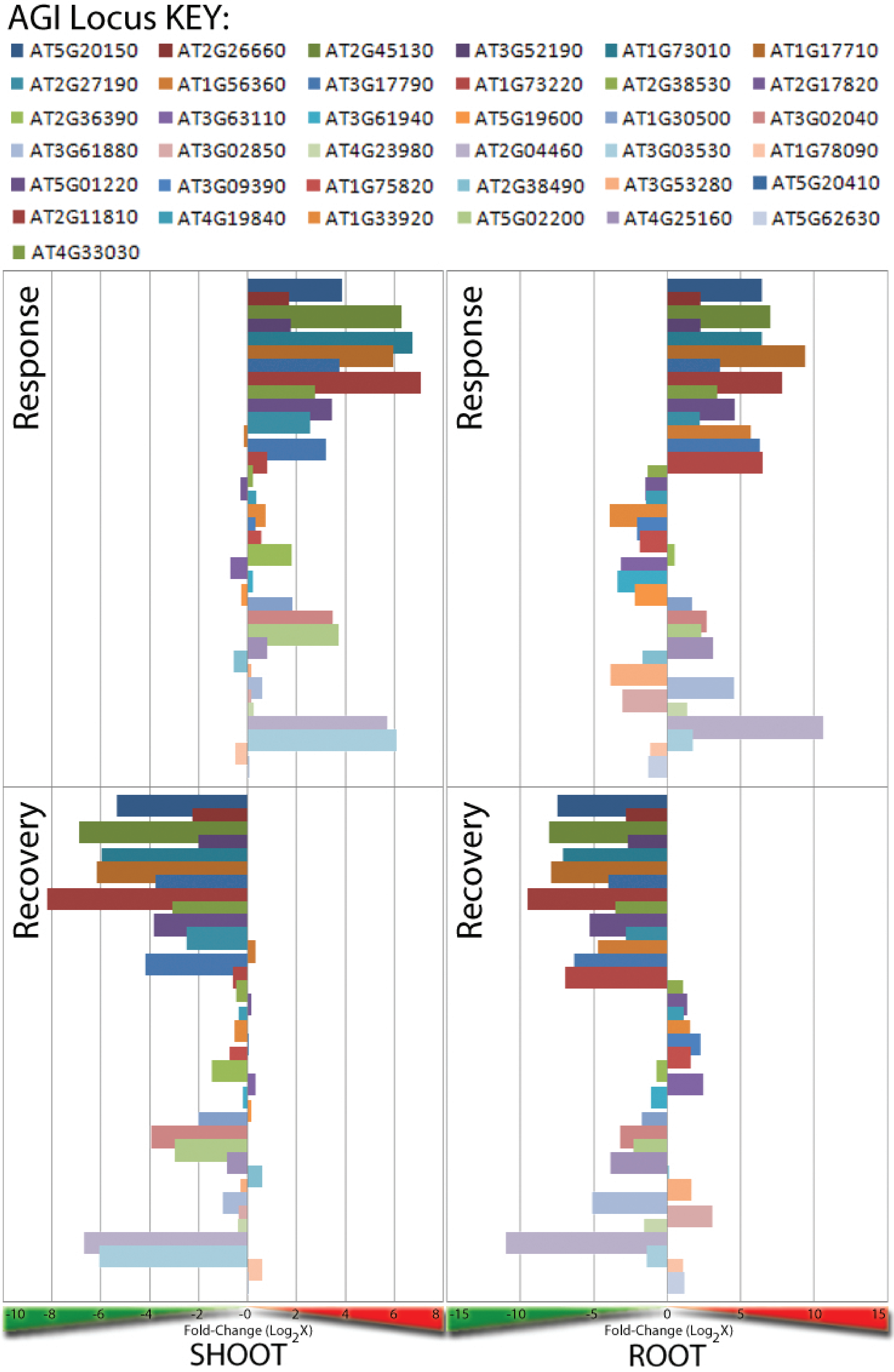

Supp. Figure: qPCR Results confirming 37 loci selected from the top differentially expresed genes known to respond to P_i starvation
