# Supplementary material for: The response and recovery of the Arabidopsis thaliana transcriptome to phosphate starvation
Source: BMC Plant Biol. 2012 May 3;12:62. doi: 10.1186/1471-2229-12-62 (PMC3520718; doi:10.1186/1471-2229-12-62)
Supplement: Additional file 7 — Gene response and recovery class list, categorizes genes into 1 of 9 response-and-recovery classes for roots and shoots, separately. [file 1471-2229-12-62-S7.pdf]

## RT-PCR of IPS1, mir399s, and miR827

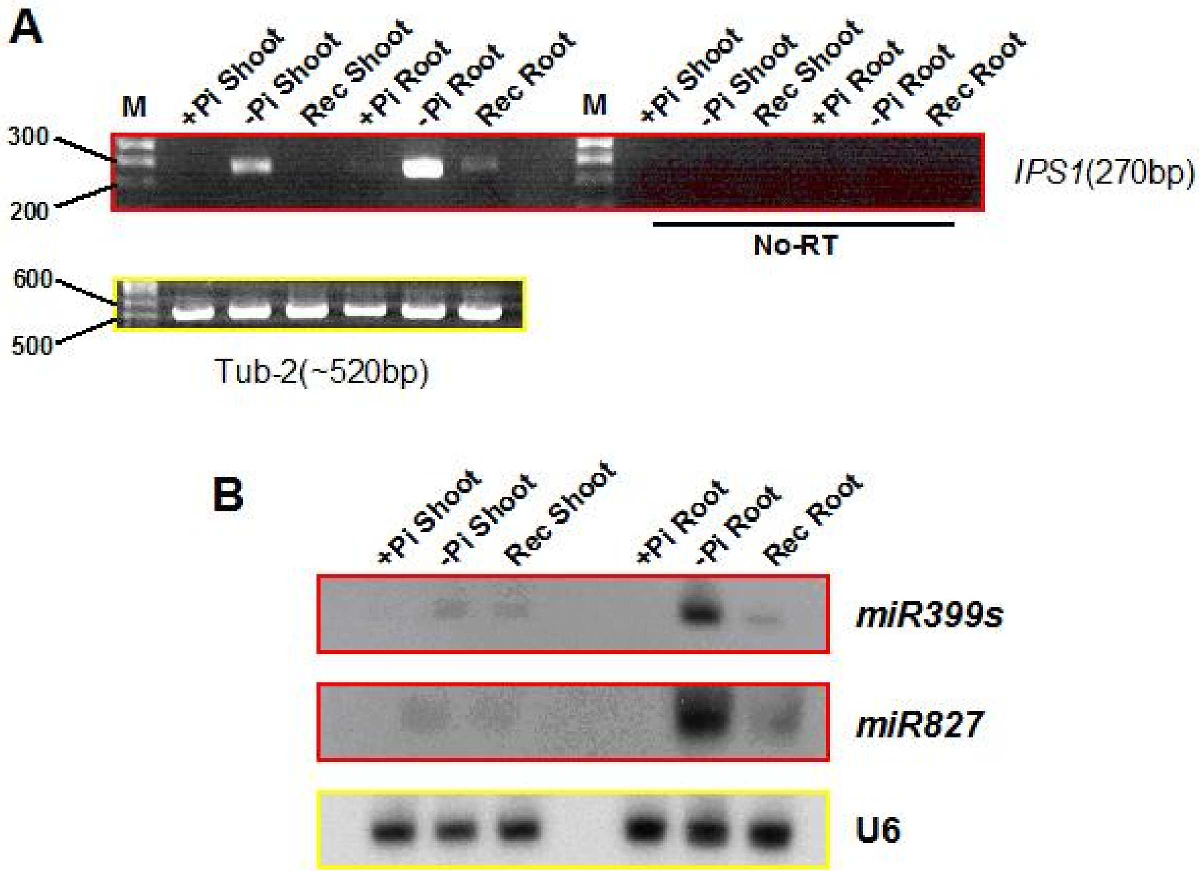


Instead of measuring free Pi content in plants, we checked the transcriptional levels of Pi-inducible marker genes to confirm when plants were exposed to enough nutrient stress. Briefly, *IPS1*, *miR399s*, and *miR827* are well-known Pi-inducible non-coding RNAs and therefore suitable marker genes. RT-PCR of *IPS1* and sRNA northern blots of *miR399*s and *miR827* was performed (supp. MarkerGenes.pdf). *Tubulin 2* for RT-PCR and U6 for sRNA northern blot were used as internal loading controls. This result indicates that our plant samples were suitable for this research even though Pi-content was not checked.

A. "+Pi":10-days full nutrient; "-Pi": 10-days Pi starved; "Rec": 10-days Pi starved followed by 3-days Pi replete; Left-with cDNA;Right-"No RT"-no cDNA.
