# Supplementary material for: The response and recovery of the Arabidopsis thaliana transcriptome to phosphate starvation
Source: BMC Plant Biol. 2012 May 3;12:62. doi: 10.1186/1471-2229-12-62 (PMC3520718; doi:10.1186/1471-2229-12-62)
Supplement: Additional file 9 — Materials and methods for Bioinformatics, the computational portion of the materials and methods section. [file 1471-2229-12-62-S9.doc]

# Supplementary Materials and Methods

# Analysis Pipeline

| Experiments were designed to complement prior research in a non-redundant manner: current knowledge was extended by simultaneous analysis of root and shoot organs, and response and recovery gene expression patterns. The experimental design and analysis pipeline is shown to the right and described below:  Experiments were designed around 3 time points; namely, mock P_i_^starv^ (M), treatment P_i_^starv^ (T), and recovery from T (R). Samples for each time-point were collected in triplicate from two broad tissue groups, shoot and root – see plant material and growth conditions in the main manuscript for further details. Thus, a total of 18 samples were collected, including biological replicates. Enough plant material was collected from each of the 18 samples to purify mRNA libraries for hybridization to both micro- and tiling-array chips supplied by Affymetrix. Chip readings were confirmed by qRT-PCR. Thus, the relative abundance of mRNA species in each replicate was queried using 2 separate and yet complementary technologies: Affymetrix-GeneChip (ATH1), and Affymetrix-TilingArray (Tiling 1.0R). Thus, the sum-total of high-throughput measurements amounted to 18 GeneChips (3^Replicates^ x 3^Time-Points^ x 2^Tissues^), and 18 TilingArrays (3^Replicates^ x 3^Time-Points^ x 2^Tissues^).  Data was analyzed and interpreted in a step-wise approach. Initial quality control (step 1) is followed by gene classification (step 2) and comparison between high-throughput platforms (step 3). Next, results are compared to current knowledge (step 4) in order to contextualize results and further aid in identification of novel results (step 5). Finally, many stress-responses are known to interact with one another and so an analysis of cross-talk is performed (step 6). | 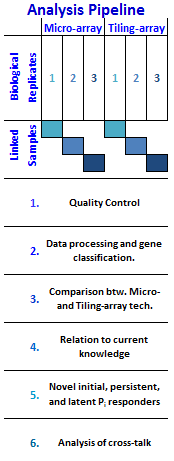 |
| --- | --- |

# Micro-array Quality Control (standard chip intensity checks)

Quality control metrics generally attempt to represent/detect differences between chips in terms of probe intensities. Detection of faulty chips and biological relevance is left to human judgment.

The following table lists standard quality control checks that were conducted for all 18 ATH1 Affymetrix arrays:

| Distributions of chip intensities were plotted and checked for abnormal behavior between chips. The ‘R’ statistical environment was used to produce the curves and box-and-whiskers plots to the right. | 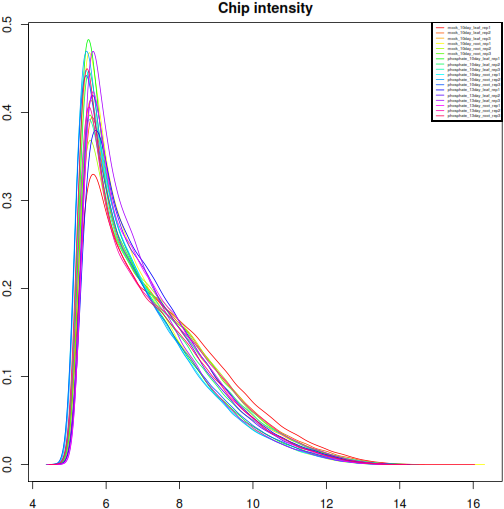 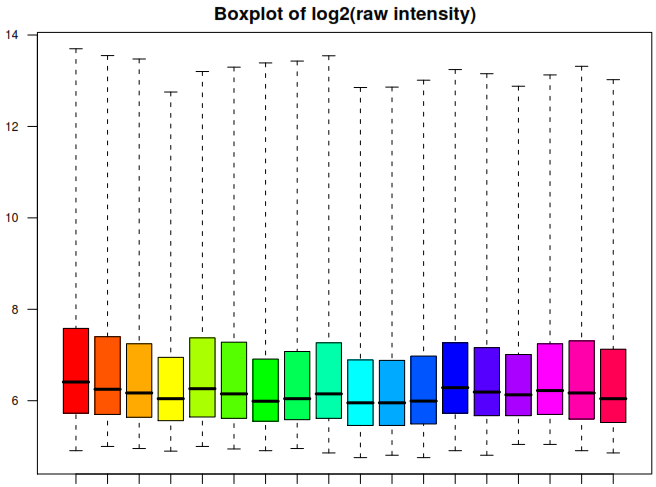 |
| --- | --- |
| Synthetic MA plots are used to determine whether or not chips contain many aberrantly over- or under-expressed genes. The ‘R’ statistical environment was used to produce the plots to the right. Each of the 18 micro-arrays were compared to a synthetic reference chip representing the median of all intensities, of which two examples are shown to the right. | 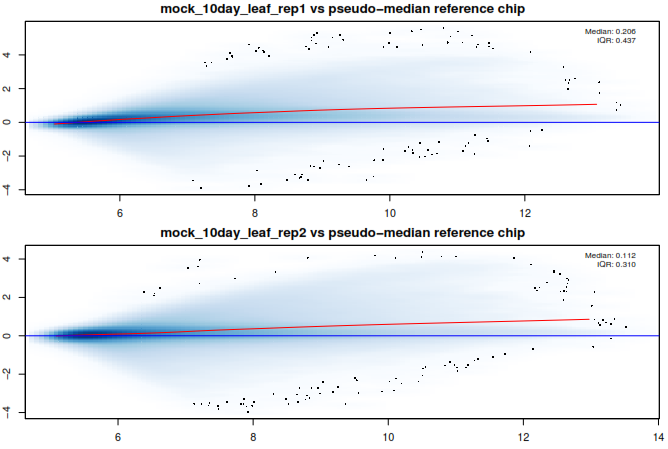 |
| RNA degradation plots measure any bias present within probe sets, between chips. The ‘R’ statistical environment was used to produce the curves to the right. Each curve represents an averaging of probes across probe-sets from 5’-3’ (x-axis) from a single ATH1 chip. | 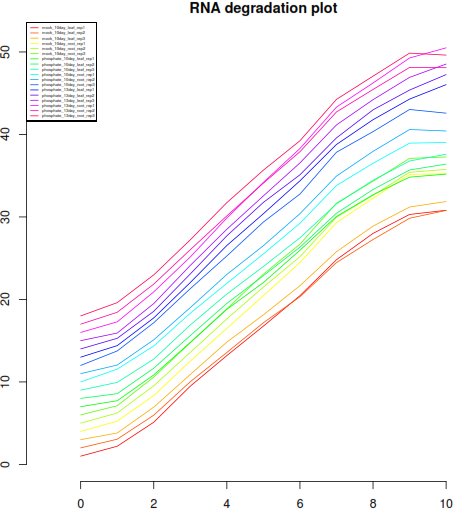 |
| We fitted a basic gene expression probe level model to the data and plotted the:   - - Relative Log Expression (RLE, top) - to check that the majority of gene expression values do not change significantly.   - Normalized Un-scaled Standard Error (NUSE, middle) - similar check to RLE but more sensitive to poor quality, NUSE plots the standard error.   - Synthetic chip images (bottom) -highlighting significantly expressing probes in order to detect any chip printing artifacts. Sample of 4 chips are given to the right.   The ‘R’ statistical environment was used to produce each image to the right. | 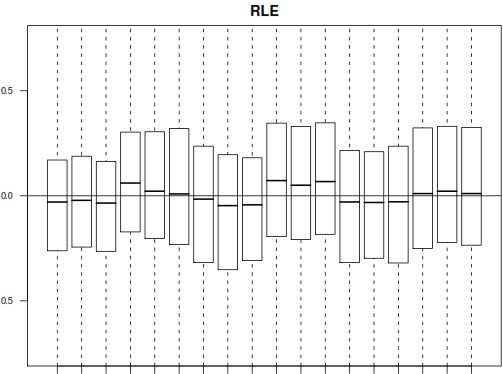 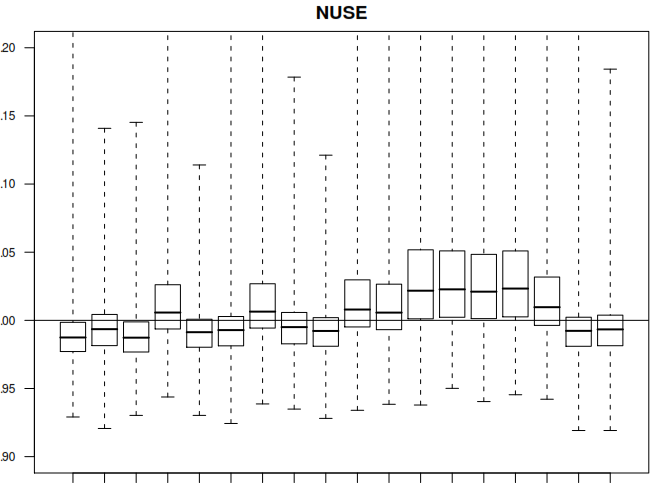  **Synthetic Chips (showing 4 of 18)**  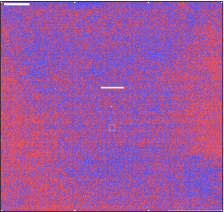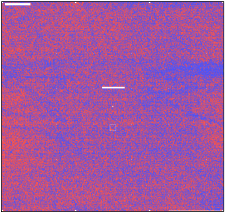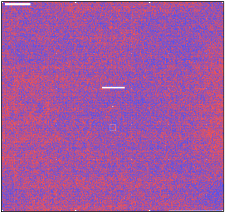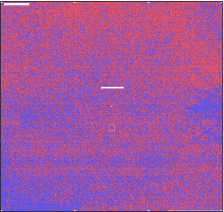 |

# Micro-array Quality Control (principal component checks)

The primary concern of any principle component analysis (PCA) is to attempt to reduce the complexity of data into as few variables as possible without losing information. Ideally, these variables should explain as much of the data as possible. To accomplish this, PCA solves for variables that describe the most variance within the dataset based on ranking methodology. Generally the top three ranked principle components (PC) can account for more than 90% of the variance within most data-sets.

A useful property of the PCA analysis is the ability to compare complex multi-dimensional data by plotting the first two, or three, PCs against one another. Each PC is composed of loading values (LVs) related to the data's original dimensions; in this case one micro-array chip represented a single dimension of probe intensities. Thus, by plotting the first few PCs against one another it is possible to visualize and assess the relationship between each of the original dimensions (or, ATH1 chips). This analysis was conducted using functions in the ‘R’ statistical environment.

# Background Correction, Normalization and Summarization

All preprocessing steps were performed using the Bioconductor package, "affy", from the ‘R’ statistical environment [1]. Specifically, background correction was performed as described by Irizarray et al. (2003) [2]. Quantile normalization was performed as described by Bolstad et al. (2003) [3]. Finally, probes were summarized into single gene expression (probe-set) values on a per chip basis using the median-polish algorithm (Tukey (1977) [4]) to fit a multi-chip probe-level model (PLM) previously used by Irizarray et al. (2003). As part of the PLM model fitting, the final gene expression values are in log_2_ scale.

# Classification of Differentially Expressed Genes

Once data was normalized there were two problems. The first was how to properly define whether or not a particular gene was being differentially expressed. The second was that once genes were determined to be differentially expressed what was the nature and biological meaning of their change in gene expression.

Of the three experiment types {Mock-starvation (**M**); Treatment-starvation (**T**); starvation-Recovery (**R**)}, two relationships were extracted to elucidate on the kinetics of genes expressing between experiments in response to P_i_^starv^. These relationships are **T**:**M** and **R**:**T**, describing the change in gene expression from **M**ock to **T**reatment and then from **T**reatment to **R**ecovery, respectively.

Here, for each relationship {**T**:**M**; **R**:**T**} we defined a gene to be differentially expressed if the Bonferroni corrected *p-value* was less than 0.001, as ascertained by the eBayes approach implemented under the ‘limma’ package in the ‘R’ statistical environment [5, 6]. Fold-change was considered during post-analysis on a per-case basis in order to avoid false-negatives.

It follows that, given a threshold cutoff, genes can either be up-regulated, down-regulated, or unchanged for any given relationship. Furthermore, these 3 states in the context of two relationships {**T**:**M**; **R**:**T**} translate to there being nine separate response-and-recovery classes that may be used to group differentially responsive genes. Classification was performed separately for root and shoot tissues thereby classifying each gene twice – separately for each organ. Thus, a total of 81 (9x9) combinations exist that, once considered together, describe the systemic plant response to P_i_^starv^.

# Significance analysis of functional annotations for differentially expressed genes

It stands to reason that genes known to respond to P_i_^starv^ would show differential expression within our data. Furthermore, these genes would be annotated as such within publicly available ontology's and pathway databases. A functional annotation analysis of differentially expressed genes would highlight the biological relevance and quality of our microarray experiments and further aid in identifying putative candidates.

To test for significant functional annotations we used the Gene Ontology-SLIM (GO-SLIM) and ARACYC pathway databases. The databases were analyzed separately using the following methodology: Significance scores were calculated using the right-sided non-parametric Fisher's Exact test. For each annotation/term, a Null Hypothesis (HO) was tested such that the proportion of genes annotated by a term and displaying differential expression is equal-to or less-than the proportion of differentially expressed genes. The *p-value* was corrected (Bonferroni correction) by a factor equal to the number of terms within the database. If any term resulted in a *p-value* less than 0.05, the HO was rejected.

Separately, for known and novel genes alike, we clustered differentially regulated genes by their functional annotations. Data was prepared by indexing all functional annotations and then annotating each differentially expressed gene with the index of its associated functional annotations. This was represented by a 2 dimensional matrix containing X treatments and Y genes. Genes were then clustered using tMev software [7, 8]. Clusters were then examined and curated in detail, by hand, as shown in Figures 3 and 4 in the main text.

# Comparison of micro- and tiling-arrays by correlation

In order to compare the two platforms, we used correlation of fold-change as a metric. Having already determined expression values for micro-array probe-sets we limited our analyses to those probe-sets identified as significantly changed. This was done in order to reduce noise caused by basally regulated probe-sets. Thus, we mapped each significantly changed probe-set to an mRNA region annotated by TAIR8. We then collected all normalized intensity values from tiling-array probes encompassed by this annotated region. Median-polish, the same method used in the processing of micro-array probe-sets, was used to obtain a single representative intensity value for the tiling-array region. Once this was performed for all significantly altered probe-sets we calculated the correlation coefficient between micro- and tiling-array for response and recovery in root and shoot organs.

# Determining interaction between several AtGenExpress treatments

Genes known to respond to P_i_^starv^ are also known to respond to such stimuli as sucrose [9, 10], cold [11], heat-shock [11], drought [11], and hormone treatments [12]. However, this knowledge was primarily assessed on a per-locus basis and not yet thoroughly investigated from a genome-wide approach. Although, Muller et al. (2007) showed that a genome-wide investigation of sucrose-P_i_ interaction can be a useful tool for discerning gene roles in P_i_^starv^ [10]. Thus, we designed a simple methodology to ascertain the degree of interaction between P_i_^starv^ responsive loci and several treatments studied by the AtGenExpress initiative. The following table lists the 21 treatment conditions from AtGenExpress that we utilized and their respective controls:

| **Number** | **Treatment** | **Control** |
| --- | --- | --- |
| **1** | 10uM AgNO3 (ethylene inhibitor) for 3h | mock treatment for 3h |
| **2** | 0.005mM Pi treatment for 10 days | 1mM Pi treatment for 10 days |
| **3** | 0uM Sulphate for 1 day | 1500uM Sulphate for 1 day |
| **4** | 150mM Sulphate for 1 day | 0mM NaCL for 1 day |
| **5** | 4 degrees celcius for 1 day | Normal temp for 1 day |
| **6** | Dry air stream until 10% weight loss | Normal conditions for 1 day |
| **7** | 3hr heatshock at 38 deg. celcius | Normal conditions for 1 day |
| **8** | 300mM Mannitol for 1 day | 0mM Mannitol for 1 day |
| **9** | 10uM Methyl viologen for 1 day | 0uM Methyl viologen for 1 day |
| **10** | UVB treatment for 1 day | Normal conditions for 1 day |
| **11** | Leaf wounded by 16pin | Normal conditions for 1 day |
| **12** | *Botrytis Cinerea* inoculation | Sterile inoculum |
| **13** | 10uM ABA for 3hr, seedling | No hormone control |
| **14** | 10uM ACC for 3hr, seedling | No hormone control |
| **15** | 10uM NPA for 3hr, seedling | No hormone control |
| **16** | 10nM BL for 3hr, seedling | No hormone control |
| **17** | 3uM Brz220 for 3hr | No hormone control |
| **18** | 10uM Prohexadione for 3hr | No hormone control |
| **19** | 1uM GA for 3hr | No hormone control |
| **20** | 1uM IAA for 3hr | No hormone control |
| **21** | 10uM MJ for 3hr | No hormone control |
| **22** | 1uM Zeatin for 3hr | No hormone control |

Treatments were compared to controls in order to determine statistically significant fold-change (Bonferroni corrected *p-value* of 0.001) for all ATH1 probe-sets that were differentially regulated during P_i_^starv^ and/or recovery. Once this statistical analysis was completed for all treatments, a network was constructed relating differentially expressed genes to respective treatment conditions. Thus, the completed network represents P_i_^starv^ responsive loci participating in at least one or more additional treatment responses. Therefore, a locus in the network may be considered to interact with P_i_^starv^ and other stimuli. Either this occurs because the locus plays separate roles in different signaling pathways or, more likely, the gene represents a focus of overlapping signaling pathways (cross-talk). In either case, this methodology aids in better defining gene functionality and generating hypotheses.

It follows that loci not described within the interaction network are P_i_^starv^ specific and have not been observed as significantly changed in any of the analyzed treatments. Thus, two issues with this approach become apparent. The first is that the measure of specificity is susceptible to the addition of more data/treatments, i.e. the more treatments we study the less likely it is that we will observe P_i_^starv^ specific loci. However, this should not be viewed as a problem since the more interaction we observe the more likely it is to elucidate on gene function. The second issue is the *p-value* threshold, which if altered may have a high impact on results. A related issue is that the thresholding methodology leads to binary results, where genes are binned into either the specific or non-specific categories. Once binned, it becomes impossible to discern the difference between loci within the two categories. Thus, thresholding is not a good solution as it prevents the question of whether one locus is more or less specific than any other, and by what degree.

To solve this problem a gene-ranking solution was developed. Here we analyzed individual loci across all AtGenExpress treatments and calculated the minimum *p-value* threshold required for that locus to be classified as non-specific. Hence, loci whose minimum *p-value* approaches 1 are basally responsive across all treatments and therefore more P_i_^starv^ specific. The minimum *p-value* metric was used to rank the subset of predefined P_i_^starv^ responsive loci with respect to one another; the results of which may be seen in the main text, Figure 5.

# References

1. Gautier L, Cope L, Bolstad BM, Irizarry RA: **affy—analysis of Affymetrix GeneChip data at the probe level.** *Bioinformatics* 2004, **20:**307-315.

2. Irizarry RA, Hobbs B, Collin F, Beazer‐Barclay YD, Antonellis KJ, Scherf U, Speed TP: **Exploration, normalization, and summaries of high density oligonucleotide array probe level data.** *Biostatistics* 2003, **4:**249-264.

3. Bolstad BM, Irizarry RA, Åstrand M, Speed TP: **A comparison of normalization methods for high density oligonucleotide array data based on variance and bias.** *Bioinformatics* 2003, **19:**185-193.

4. Mosteller F, Tukey JW: *Data Analysis and Regression: A Second Course in Statistics.* Addison-Wesley; 1977.

5. Clayton D, Kaldor J: **Empirical Bayes estimates of age-standardized relative risks for use in disease mapping.** *Biometrics* 1987, **43:**671-681.

6. Smyth GK: **Limma: linear models for microarray data.** In *Bioinformatics and Computational Biology Solutions using R and Bioconductor.* Edited by Gentleman R, Carey V, Dudoit S, Irizarry R, Huber W: Springer, New York; 2005: 397-420

7. Saeed AI, Bhagabati NK, Braisted JC, Liang W, Sharov V, Howe EA, Li J, Thiagarajan M, White JA, Quackenbush J: **TM4 microarray software suite.** *Methods Enzymol* 2006, **411:**134-193.

8. Saeed AI, Sharov V, White J, Li J, Liang W, Bhagabati N, Braisted J, Klapa M, Currier T, Thiagarajan M, et al: **TM4: a free, open-source system for microarray data management and analysis.** *Biotechniques* 2003, **34:**374-378.

9. Franco-Zorrilla JM, Martín AC, Leyva A, Paz-Ares J: **Interaction between Phosphate-Starvation, Sugar, and Cytokinin Signaling in Arabidopsis and the Roles of Cytokinin Receptors CRE1/AHK4 and AHK3.** *Plant Physiology* 2005, **138:**847-857.

10. Muller R, Morant M, Jarmer H, Nilsson L, Nielsen TH: **Genome-wide analysis of the Arabidopsis leaf transcriptome reveals interaction of phosphate and sugar metabolism.** *Plant Physiol* 2007, **143:**156-171.

11. Hammond JP, Bennett MJ, Bowen HC, Broadley MR, Eastwood DC, May ST, Rahn C, Swarup R, Woolaway KE, White PJ: **Changes in Gene Expression in Arabidopsis Shoots during Phosphate Starvation and the Potential for Developing Smart Plants.** *Plant Physiology* 2003, **132:**578-596.

12. Martín AC, Del Pozo JC, Iglesias J, Rubio V, Solano R, De La Peña A, Leyva A, Paz-Ares J: **Influence of cytokinins on the expression of phosphate starvation responsive genes in Arabidopsis.** *The Plant Journal* 2000, **24:**559-567.
